# Fabaceae Flavonoids Beyond the Commonplace: A Review of Chemical Diversity, Pharmacological Activities, Mass Spectrometric Profiling and *In Silico* Insights into Their Subclasses

**DOI:** 10.3390/plants14233549

**Published:** 2025-11-21

**Authors:** Ana Rita Rodrigues de Almeida Silva Brilhante, Gabriela Ribeiro de Sousa, Ana Karoline Silva de Aquino-Vital, Natanael Teles Ramos de Lima, Ranna Beatris de Lima Souza, Thalisson Amorim de Souza, Marcus Tullius Scotti, José Maria Barbosa Filho, Josean Fechine Tavares, Marcelo Sobral da Silva

**Affiliations:** 1 Graduate Program in Natural and Synthetic Bioactive Products, Federal University of Paraiba, João Pessoa 58051-900, Brazilthalisson.amorim@ltf.ufpb.br (T.A.d.S.); mtscotti@gmail.com (M.T.S.); barbosa.ufpb@gmail.com (J.M.B.F.); marcelosobral@ltf.ufpb.br (M.S.d.S.); 2 Department of Chemistry, Exact and Natural Sciences Centre, Federal University of Paraiba, João Pessoa 58051-900, Brazil; 3 Department of Pharmaceutical Sciences, Health Sciences Centre, Federal University of Paraiba, João Pessoa 58051-900, Brazil

**Keywords:** unusual flavonoids, pharmacological properties, HPLC-MS/MS

## Abstract

Fabaceae family is recognized as a prolific flavonoid producer, including some unusual flavonoid skeletons. Although classic flavonoids have well-established biological properties in the literature, these unusual compounds remain underinvestigated. Based on that, the current study sought to explore the chemistry and biological activity of rare flavonoids in Fabaceae with a review comprising their occurrence, extraction, isolation and pharmacological potential. Additionally, the use of LC-MS applied to the study of seven subclasses (aurones, biflavonoids, coumestans, homoisoflavonoids, neoflavonoids, pterocarpans, and rotenoids) is also discussed. The review was carried out by searching for specified uniterms on SciFinder and Web of Science, covering the last ten years. In addition, to assess ADMET and drug-like properties by in silico predictions, all the substances considered in this work were compiled and organized into a curated dataset. As a result, a total of 170 flavonoid structures were included, most of which were found in the roots and leaves. In addition, a wide range of biological activities were observed, such as cytotoxic, antiviral and anti-inflammatory. Despite advancements in the use of LC-MS for bioprospection, just a few reports dedicated to the study of these compounds were found. Regarding ADMET profiles, most subclasses showed favorable bioavailability characteristics, with biflavonoids being the main exception. Together, this review highlights the significance of unusual flavonoids from the Fabaceae family, demonstrating their remarkable chemical diversity and largely, but still, unexplored pharmacological potential. These findings encourage further investigations, particularly in the fields of natural products chemistry, medicinal chemistry, and pharmacology.

## 1. Introduction

Flavonoids represent a diverse group of plant secondary metabolites, chemically characterized by the fact that they comprise 15 carbon atoms arranged in a C6-C3-C6 pattern. These compounds are distributed throughout different plant tissues, including seeds, leaves, stems, flowers, and roots; more than 10,000 distinct flavonoids have been identified to date. Based on their structural features, flavonoids can be classified into subclasses depending on modifications in the oxidation and hydroxylation patterns of the central heterocyclic ring (ring C) such as flavones, flavanones, flavonols, and anthocyanidins. In addition to their structural diversity, flavonoids perform multiple ecological roles in plants, including ultraviolet (UV) radiation protection, pathogen defense, and pollinator attraction [1,2,3].

Ranked as the third largest family among angiosperms [4], Fabaceae species exhibit a remarkable capacity for flavonoid biosynthesis, attributed both to their metabolic versatility and to the wide range of ecological functions carried out by these compounds [1,3,5]. The extensive diversity of flavonoids within Fabaceae makes this family a relevant model for studies on the evolution and functionality of these secondary metabolites. Nonetheless, variations beyond the canonical skeleton do occur. The activity of specific enzymes on more common flavonoid structures, such as flavones, chalcones, and isoflavones, can lead to the formation of other flavonoid subclasses. These transformations may involve the formation of new heterocyclic rings, the establishment of single or double carbon–carbon (C–C) bonds between rings A and C, or the dimerization of monomeric units.

Certain subclasses, such as aurones, coumestans, neoflavonoids, and rotenoids, are biosynthesized through more specialized pathways and tend to be restricted to specific taxa within the Fabaceae family, indicating their potential value as chemotaxonomic markers [6,7,8,9]. In addition to their taxonomic role, the structural diversity of the flavonoids to which these subclasses belong has also been associated with a wide range of biological activities, including anti-inflammatory, cardioprotective, antibacterial, antifungal, antiviral, and antitumor properties, with many of these compounds exhibiting low toxicity [1,10,11].

Liquid chromatography coupled with mass spectrometry (LC-MS) is especially advantageous for the analysis of flavonoids, since these compounds have an ideal polarity range to exploit the analytical versatility of the technique, optimizing chromatographic separation. Furthermore, their chemical structure favors the main ionization methods used in mass spectrometry, such as ESI (electrospray ionization) and APCI (atmospheric pressure chemical ionization) [12]. Although flavonoids exhibit structural characteristics that favor their analysis by LC-MS, resulting in fragmentation patterns that are well described in the literature, the broad structural diversity of the class still requires further studies to establish more targeted analytical approaches for this class of metabolites [13].

Considering the chemotaxonomic and pharmacological importance of these uncommon flavonoids, this review provides a comprehensive overview of seven flavonoid subclasses reported in Fabaceae from 2015 to 2025. The discussion includes extraction, isolation methodologies, as well as an in-depth analysis of the pharmacological activities associated with these compounds over the past ten years. In addition, in view of the wide contribution of LC-MS in recent decades for the analysis and discovery of natural phenolic derivatives, an examination of the analytical parameters and characteristic fragmentation patterns was carried out. This work sought to guide future research into Fabaceae flavonoids and support the exploration of their full pharmacological and chemotaxonomic potential.

## 2. Methodology

### 2.1. Data Collection

To organize the data presented in this review, searches were conducted using specific sets of keywords, combining the terms “Leguminosae” and “Fabaceae” with each class of uncommon flavonoids selected in the study separately. These classes included “aurones,” “neoflavonoids,” “biflavonoids,” “rotenoids,” “coumestans,” “pterocarpans,” and “homoisoflavonoids.” Searches were conducted in the SciFinder and Web of Science databases, covering the period from January 2015 to December 2025. Review articles, flavonoids from subclasses other than those mentioned, articles without compound isolation, and publications outside the selected period were excluded. A total of 52 articles (Tables S1–S7, Supplementary Materials) were selected, from which the chemical structure, species description, solvent used for extraction and purification, plant tissue, and pharmacological tests were then classified by compound subgroup. References cited in Tables S1–S7 are included exclusively in the Supplementary Materials.

### 2.2. Fabaceae Flavonoids ADMET and Drug-likeness

Prior to analysis, the compounds were sorted by subclass and grouped to compose a dataset containing all the 170 flavonoid structures. After that, they were converted to Molecular Input Line Entry System (SMILES) and subjected to Drug-likeness and ADMET molecular descriptors generation using Alvadesc, version v2.0.016, and SwissADME. Based on the results, a unified matrix was obtained and used as input data in Unscrumble software, version 9.7, then a PCA analysis was carried out in order to illustrate the chemical space comprised by the flavonoids and identify patterns within the chemical dataset, as previously described [14].

## 3. Biosynthesis

The biosynthetic pathway of flavonoids predominantly begins with the shikimic acid pathway. In this pathway, phenylalanine, an essential precursor of the phenylpropanoid biosynthetic pathway, is converted to cinnamic acid by the action of the enzyme phenylalanine ammonia lyase (PAL), which removes the amino group from the molecule. Subsequently, the enzyme cinnamate-4-hydroxylase (C4H) catalyzes the hydroxylation of trans-cinnamic acid, producing p-coumaric acid, which binds to coenzyme A, forming 4-coumaroyl-CoA [15,16,17]. This intermediate condenses with three malonyl-CoA units through the action of different enzymes, such as chalcone synthase, chromone synthase, and stilbene synthase, resulting in the formation of chalcones, chromones, and stilbenes, respectively, which are key precursors for the formation of different subclasses of compounds. Although the biosynthesis of classic flavonoids is well established, many gaps remain in the study of the biosynthesis of more unusual flavonoids. Isoflavone synthase (IFS) is the key enzyme responsible for the migration of ring B from the C2 position to the C3 position of the central ring, generating the isoflavone skeleton. This structural rearrangement is relatively rare in nature, which explains its limited occurrence in plant taxa, with a notable abundance in the Fabaceae family. Isoflavones can generate other specialized subclasses: rotenoids, homoisoflavonoids, pterocarpans, coumestans. The structural complexity of these molecules results from hydroxylation, methylation and alkylation reactions that can alter the oxidation state of the heterocyclic ring or promote the formation of additional rings. The action of other enzymes, such as isoflavone reductase (IFR) and 4′-methoxyisoflavanol dehydratase (DMID) are also responsible for the chemical diversity of compounds derived from chalcones [15,16,18]. Chalcones, in turn, are the central precursor of different subclasses of flavonoids through a diverse and often unexplored enzymatic arsenal, such as aurones formed by the enzymes aureusidine synthase (AUS) and aureusidine 7-O-glycosyltransferase (A7GT) [16,17].

Despite the rapid and continuous advances in the areas of genomics and metabolomics, the complete elucidation of complex biosynthetic pathways, often mediated by a diverse enzyme arsenal, is still limited and insufficient to fully understand the formation of new metabolites or rare natural products. The biosynthetic pathways of some of these subclasses, such as coumestans and homoisoflavonoids, which are derived from isoflavonoids, do not have well-defined biosynthetic pathways. The same applies to neoflavonoids, mainly due to their structural rarity. These compounds, characterized by an aryl bond on carbon 4 of the central ring (as opposed to the carbon 2 typical of flavonoids), can result from enzymatic modifications of the flavanone or isoflavonoid core, including oxidative rearrangements or prenylation reactions followed by cyclization. Meanwhile, the classic study by Ollis (1966) [19] also proposed that neoflavonoids could be formed from the acylation of 4-coumaryl-CoA; although this hypothesis remains speculative and lacks up-to-date biochemical validation.

In this perspective, some aspects of synthetic biology have integrated knowledge of computational biology and biotechnology with the aim of unraveling the complexity of metabolic processes associated with the biosynthesis of natural compounds. Among the strategies adopted are the manipulation of the expression of key genes in the synthesis of enzymatic proteins, the development of technologies based on artificial intelligence and the use of in silico approaches to predict biosynthetic routes [20,21]; therefore, contributing to understand the highly specialized biosynthetic routes that are responsible for the great structural diversity of natural products.

## 4. Extraction and Isolation

**Figure 1:**
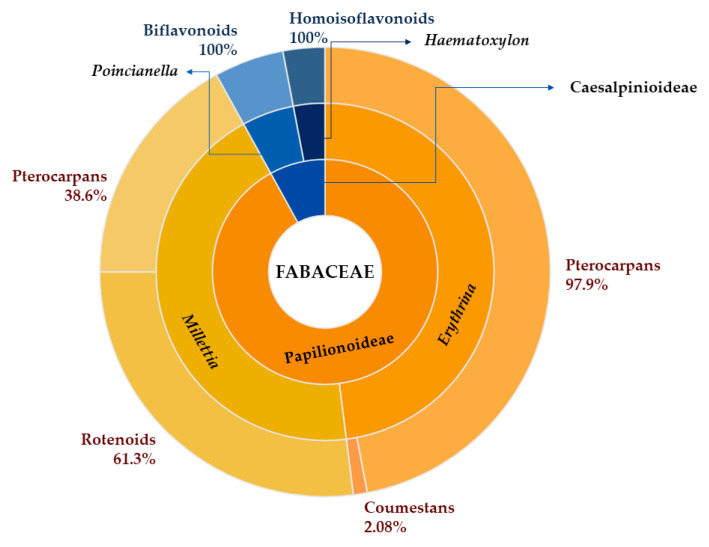
Main genera of plants rich in unusual flavonoids, grouped by subfamily (Papilionoideae and Caesalpinioideae) and percentage of occurrence by class of compounds produced by the main genera reported in this review.

The unusual flavonoid compounds reported in this work are distributed among a total of 50 species in 28 genera belonging mainly to the subfamily Papilionoideae, with 25 distinct genera and the remainder belonging to the subfamily Caesalpinioideae. Among the genera of the Papilionoideae subfamily, *Erythrina* and *Millettia* are particularly notable in terms of the occurrence of species producing these unusual flavonoid compounds, while *Poincianella* and *Haematoxylum* are the largest genera of producing species belonging to the Caesalpinioideae family (Figure 1). Interestingly, in the main genera of the Papilionoideae subfamily there is no pattern of occurrence between the classes of compounds as is observed for the species of the Caesalpinioideae subfamily which produce mostly biflavonoids and homoisoflavonoids, demonstrating the chemical diversity possibly related to the complex chemotaxonomy of this subfamily.

**Figure 2:**
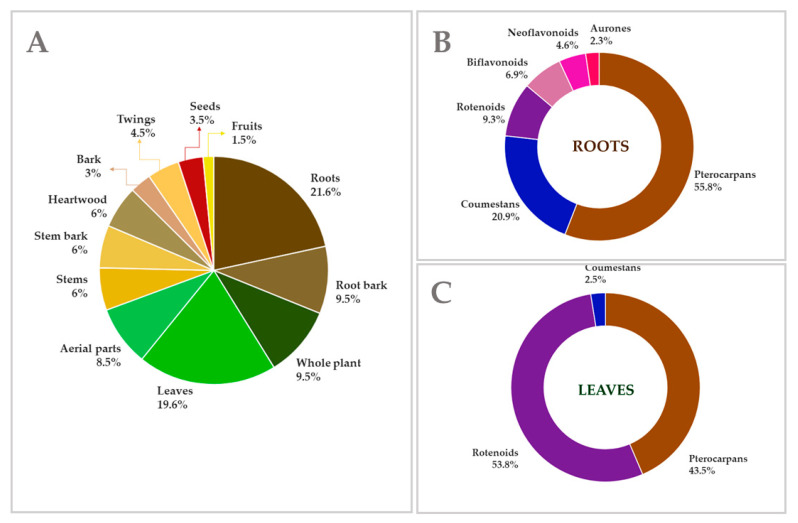
(**A**) Percentage distribution of plant parts used as sources of the isolated metabolites, showing the total number of substances per plant tissue. (**B**) Distribution of the most frequently found flavonoid subclasses among the compounds isolated from roots. (**C**) Distribution of the most frequently found flavonoid subclasses among the compounds isolated from leaves.

These compounds were found in various plant tissue, including roots, leaves, fruits, and seeds. However, the majority of these compounds were obtained from the roots and root bark, with a total of 62 substances occurring, followed by the leaves (39), stem and stem bark (24), whole plant (20), and aerial parts (17) (Figure 2). The majority of the compound classes are found in the roots and root bark, especially the pterocarpans (38), although they are also distributed throughout all plant components. Interestingly, the only class that was not reported in the roots were the homoisoflavonoids, which were mostly found in the stem or heartwood (5) of the species studied. On the other hand, rotenoids, aurones and coumestans were obtained mainly from the leaves (21), aerial parts (10) and whole plant (10), respectively.

The predominance of these compounds in the roots is associated with the involvement of flavonoids in multiple functions in root-rhizosphere signaling. They can be involved in stimulating or inhibiting rhizobial nodule gene expression and chemoattraction of rhizobia towards the root, stimulating the germination of mycorrhizal spores and the branching of hyphae, modulating allelopathic interactions between plants, as well as favoring the uptake of nutrients from the soil [22,23]. Compounds of the pterocarpan class, for example, are known as phytoalexins and are responsible for plant defense against pathogens. Studies also suggest that in the interaction between plant-microbe these compounds may not only remain accumulated in the roots but are also secreted by the roots, preventing possible future infection [24,25].

These data suggest a complex enzymatic arsenal present in the roots, allowing the production of compounds from various classes of rare flavonoids. On the other hand, a biosynthetic specificity is also observed in the leaves, given the majority occurrence of rotenoids in this tissue. In addition, they reinforce the importance of knowledge about the possible biological and chemical interactions present in nature as a stimulus for the production of new bioactive compounds in the different tissues of these species, which can be investigated through advanced metabolomics studies.

In general, the extraction and separation of flavonoids from leguminous plants follows a consistent methodological pattern across the different subclasses. Most compounds reported in the reviewed studies were isolated from ethyl acetate and n-butanol fractions, obtained by liquid–liquid partitioning of crude methanolic or ethanolic extracts. The purification processes are predominantly carried out by column chromatography (CC) using silica gel, Sephadex LH-20, and polyamide as stationary phases, often combined with gradient elution systems of increasing polarity (hexane–ethyl acetate–methanol). For prenylated or methoxylated flavonoids, which generally exhibit higher lipophilicity, reversed-phase (RP-18) columns and preparative HPLC have proven effective for achieving purified fractions. This combined approach, integrating selective solvent partitioning with conventional chromatographic techniques, has been particularly useful for the isolation of structurally complex flavonoids, such as pterocarpans, homoisoflavonoids, and neoflavonoids, which often coexist in matrices that are rich in structural isomers. Therefore, the use of intermediate-polarity solvents and high-efficiency chromatographic columns represent a key strategy for the targeted separation of rare and structurally diverse flavonoids within the Fabaceae family.

### 4.1. Aurones

Aurones are a subclass of phenolic compounds named after the Latin word *aurum* (gold), due to the yellow pigmentation they confer to the plants in which they are found [26]. These compounds feature a 6:5 benzofuranone core linked to a 2-aryl group and are structurally related to flavones, differing by the presence of a five-membered central ring instead of six. Although less studied than other flavonoid subclasses, aurones have recently attracted attention due to their promising pharmacological properties [6].

**Figure 3:**
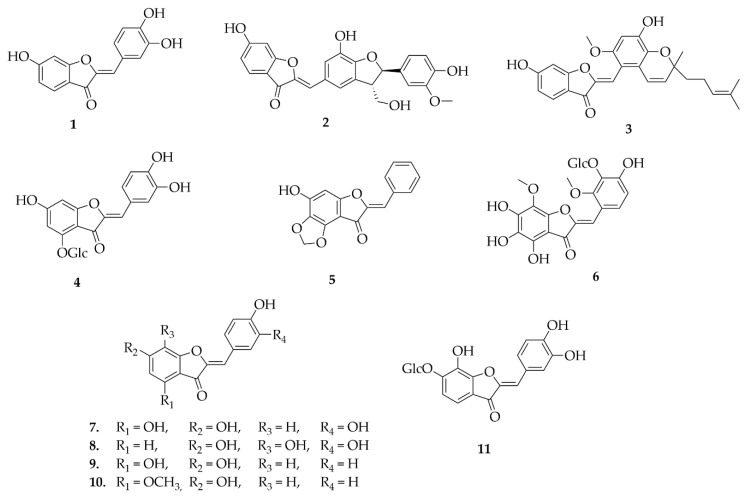
Aurones reported in this review.

Only eleven (Figure 3) compounds of the aurone class have been reported in the Fabaceae family, with distribution restricted to the Papilionoideae subfamily. The occurrence of these compounds in this subfamily includes three genera: *Astragalus*, *Cassia* and *Sophora*. The substances were mostly obtained from ethanolic and methanolic extracts of the aerial parts and roots. The compound sulphuretin (**1**) was obtained from a methanolic extract of the aerial parts of *Cassia nomame.* From the ethanolic extract obtained from the aerial parts of *Sophora japonica*, the compounds altilisin I (**3**), (*Z*)-4-*O*-*β*-*D*-glucopyranosyl-7,3′,4′-trihydroxyaurone (**4**), *Z*)-3′-*O*-*β*-*D*-Glucopyranosyl-4,5,6,4′-tetrahydroxy-7,2′dimethoxyaurone (**5**), (*Z*)-4,5-methylenedioxy-6-hydroxybenzene-2[(2′,3′,4′-trimethoxy)-2-methyl-2(4-methyl-3-penten-1-yl)-2H-1-benzopyran-5-yl) methylene]-3-benzofuranon (**6**), cephalocerone (**7**), (*Z*)-6,7,3′,4′-Tetrahydroxyaurone (**8**), (*Z*)-4,6,4′-Trihydroxyaurone (**9**), (*Z*)-4-Methoxy-6,4′-dihydroxyaurone (**10**), (*Z*)-6-*O*-*β*-*D*-Glucopyranosyl-7,3′,4′-trihydroxyaurone (**11**) [27]. Astrernestin (**2**) was the only compound obtained from the methanolic extract of *Astragalus ernestii* [28,29].

### 4.2. Biflavonoids

**Figure 4:**
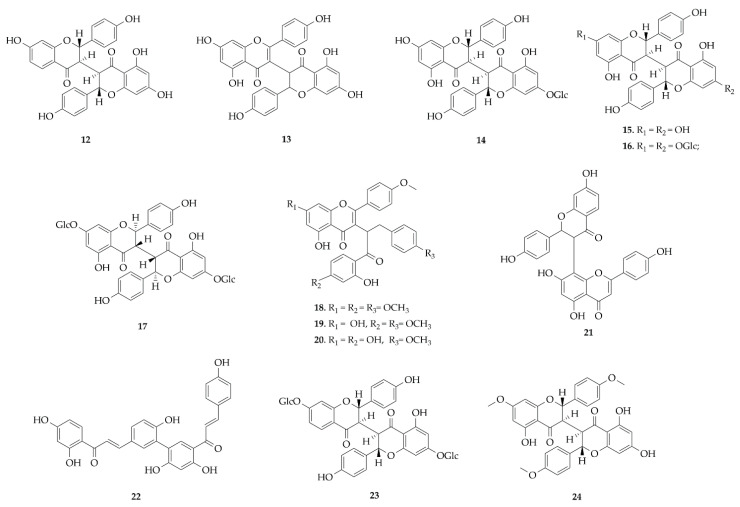
Biflavonoids reported in this review.

Biflavonoids are structurally characterized by dimerization of different subtypes of flavonoids, such as flavone, flavanone, flavonol, chalcones or mixtures of these, binding in different positions, and may or may not be glycosylated [30]. In this report, a total of 13 distinct compounds were identified in Fabaceae, distributed in the genera *Poincianella* [31] and *Ormocarpum* [32,33], belonging to the subfamily Caesalpinioideae and Papilionoideae, respectively. Most species produce biflavonoids of the IC3-IIC3 linked type, such as chamaejasmine derivatives (**8**), with a total of nine occurrences in species of the *Ormocarpum*, like *O. kirkii* and *O. sennoides* subsp. *zanzibaricum* [32,33]. Some studies have shown that species of the genus *Ormocarpum* produce biflavonoids with connectivities of (3 → 3), (3′′ → 3), (3 → 3′′) and (3 → 2′′) between the flavonoid skeletons, which may suggest a specificity in the biosynthesis of these compounds in these species, guiding future chemotaxonomic investigations [34,35]. In addition, it was observed that most biflavonoids were those formed by the combination of flavones and flavanones (**12**–**17**) [32,33]. But also, biflavonoids derived from the union of chalcones (**22**) [31] were also documented in this review (Figure 4).

### 4.3. Coumestans

Coumestans constitute a class of tetracyclic heterocyclic compounds, chemically derived from the oxidation of pterocarpans and pterocarpenes. The first report of its natural occurrence was published by Govindachari and collaborators in 1956 [36], following the isolation of a coumestan from the leaves of *Wedelia calendulacea* (Compositae), which was later also isolated in the roots and seeds. However, the majority of naturally occurring coumestans currently reported in the literature are derived from species within the Fabaceae family [37]. Activities have been attributed to coumestans, including anti-inflammatory, antimicrobial, and antiestrogenic properties [7,37].

**Figure 5:**
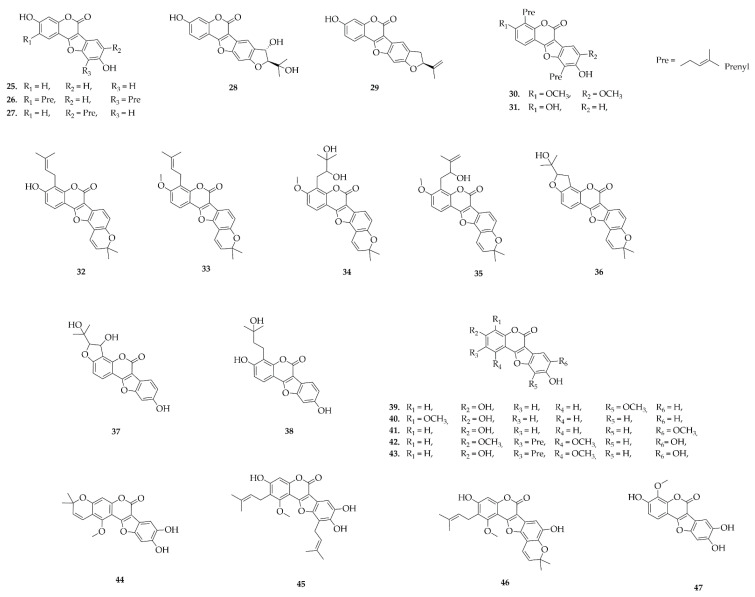
Coumestans reported in this review.

The majority of coumestans (Figure 5) in Fabaceae are found predominantly in species of the Papilionoideae subfamily, especially the genera *Pueraria*, *Erythrina*, *Tephrosia*, *Bituminaria*, *Hedysarum*, *Flemingia*, *Psoralea*, *Glycine*, *Pongamia*, *Lespedeza*, *Dalbergia*, *Glycyrrhiza* and *Butea*. Among all the compounds isolated, there was a concentrated distribution in the whole plant and roots, while compounds obtained from seeds and leaves were less representative. The use of ethanol was predominant in the extraction of the reported coumestans; however, chloroform and methanol were also able to extract these types of molecules.

In addition, some molecules have shown wide distribution among different species and tribes, like *Phaseoleae*, *Psoraleeae*, *Millettieae*, *Desmodieae* and *Dalbergieae*. Cumestrol (**25**), for example, was found in the roots of *Pueraria mirifica*, in the leaves of *Bituminaria morisiana* and in *Glycine tabacina* [38,39,40]. Other molecules similar to coumestrol, but with cyclized prenyl (Glytabastan C-G), were also isolated from whole plant of *Glycine tabacina*. Sigmoidin K (**26**) was isolated from the roots of species of the genus *Erythrina* (*E. subumbrans*) [8]. These findings reinforce the structural diversity and variable distribution of coumestans within the Fabaceae family.

### 4.4. Homoisoflavonoids

**Figure 6:**
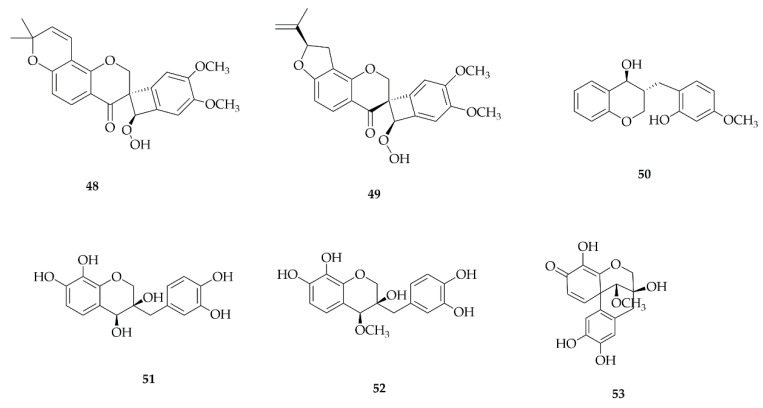
Homoisoflavonoids reported in this review.

Homoisoflavonoids (Figure 6) represent a relatively uncommon subclass of flavonoids, characterized by the presence of an additional carbon atom connecting the B and C rings of the flavonoid skeleton. In the Fabaceae family, they have been reported in the genera Crotalaria, Caesalpinia, and Haematoxylum, and have been isolated from seeds, stems, and heartwood using solvents of different polarities (Table S4) [41]. Among the reported species, Haematoxylum campechianum (Caesalpinioideae) stands out for producing naturally occurring homoisoflavonoids such as hematoxylol (**51**), 4-*O*-methylhematoxylol (**52**), and hematoxin (**53**), which were isolated from the heartwood using methanol [42]. These compounds share a common homoisoflavonoid scaffold derived from hematoxylin and illustrate the structural diversity that can arise through natural methylation processes. 

In general, homoisoflavonoids have been isolated from hydroethanolic, ethanolic, and methanolic extracts, with some cases using acidified aqueous extractions. Their distribution in woody and reproductive tissues suggests a protective ecological role and potential chemotaxonomic significance.

### 4.5. Neoflavonoids

**Figure 7:**
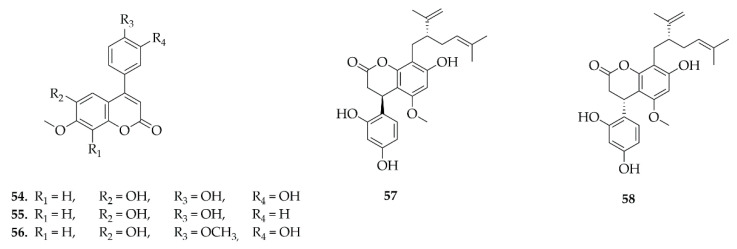
Neoflavonoids reported in this review.

Neoflavonoids differ from the classical flavonoid skeletons due to the attachment of the B ring to position 4 of the C ring, rather than position 2, resulting in the basic structure of 4-phenylcoumarins. These compounds have been reported in the Fabaceae and Moraceae families and, despite their limited occurrence, are notably present in the *Dalbergia* genus. Here, the compounds were isolated strictly from the Papilionoideae subfamily (Figure 7), which includes two genera: *Dalbergia* and *Sophora*. The substances were obtained from ethanolic extracts of the roots and heartwood. The compounds 3′-hydroxymelanettin (**54**), Melanettin (**55**), and Melannein (**56**) were found in the ethanolic extracts of the heartwood of *Dalbergia melanoxylon* [43,44]. Sophoraneoflavonoid A and B (**57** and **58**) were isolated from the ethanolic extract of *Sophora flavescens* roots [45].

### 4.6. Pterocarpans

**Figure 8:**
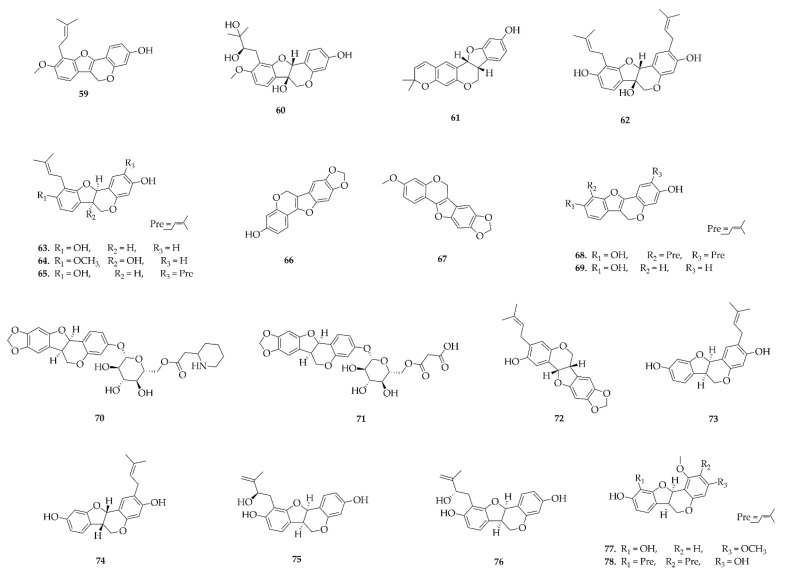
Pterocarpans reported in this review.

**Figure 9:**
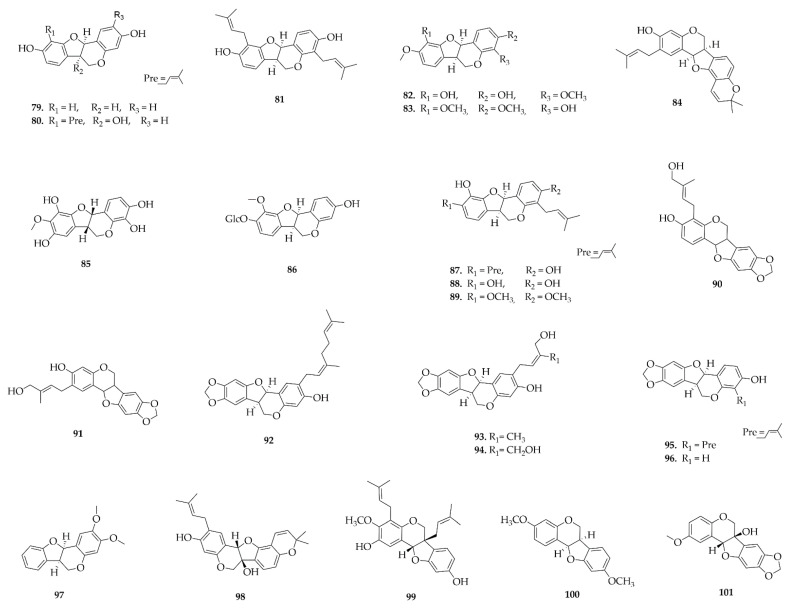
Pterocarpans reported in this review (continued).

**Figure 10:**
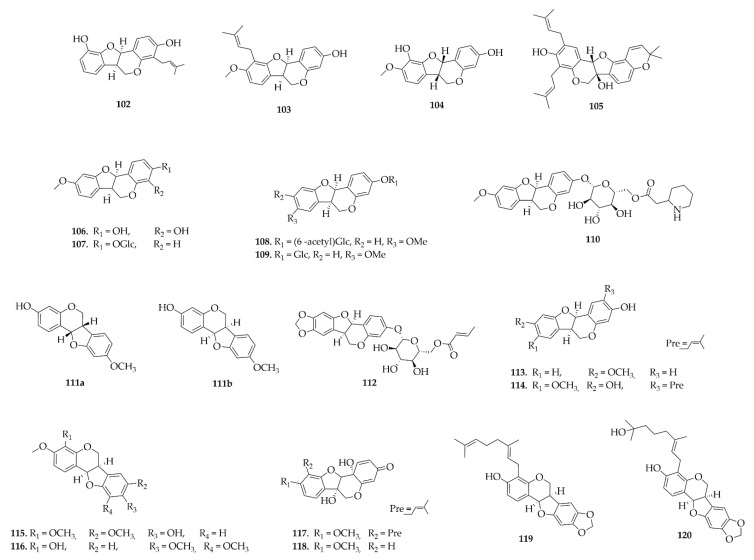
Pterocarpans reported in this review (continued).

**Figure 11:**
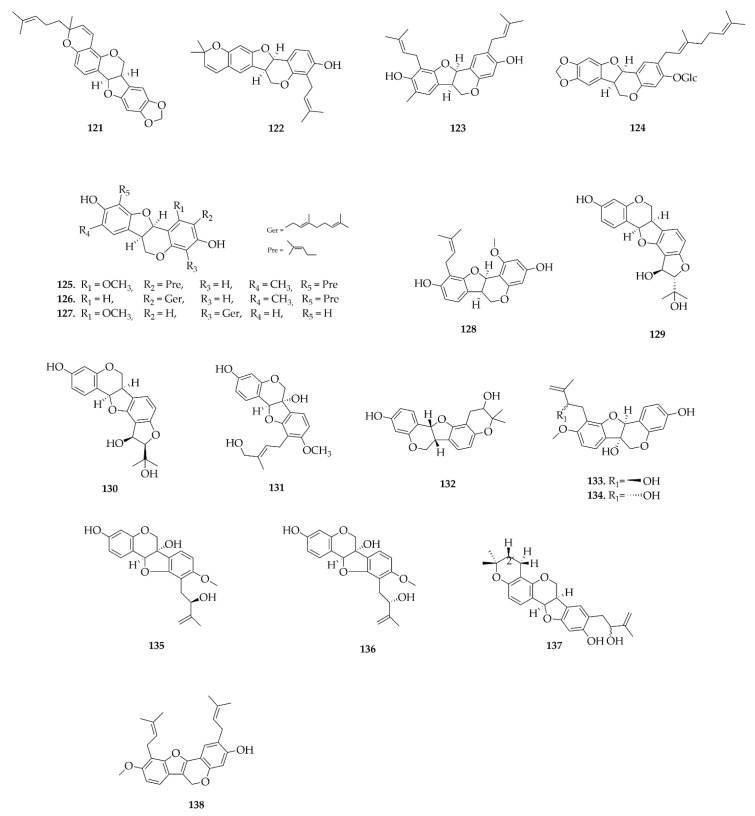
Pterocarpans reported in this review (continued).

Pterocarpans were the most frequently reported class in this review, a total of 81 different pterocarpans (Figure 8, Figure 9, Figure 10 and Figure 11) have been reported and distributed in the Papilionoideae subfamily, suggesting a distribution restricted to this subfamily during this period. The diversity of genera in the Papilionoideae subfamily from which compounds of the pterocarpan class have been isolated is vast, but the genera with the highest occurrence of these compounds were *Erythrina* and *Milletia*, with 47 and 44 isolated compounds, respectively.

The compounds were isolated mainly from the roots of these plants, followed by the leaves, the whole plant, the root bark, and aerial parts. The presence of these compounds is also reported, less frequently, in the stem bark, stem, and heartwood, and even more rarely in the twigs, bark, seeds, and fruits. Although the data shows a higher occurrence in the roots of Papilionoideae species, the investigation of the content of this class of compounds in other components of the plant cannot be discarded, as they are still under-explored.

As for the extracts used for these isolations, the most common were methanolic and ethanolic extracts, which allow for the extraction of components with a wide range of polarity, consequently contemplating the chemical diversity of these compounds. However, other extracting solvents can also be used which allow the targeting of a certain profile of compounds of interest, such as dichloromethane (CH_2_Cl_2_), chloroform (CHCl_3_), ethyl acetate, hexane, water, and acetone.

### 4.7. Rotenoids

**Figure 12:**
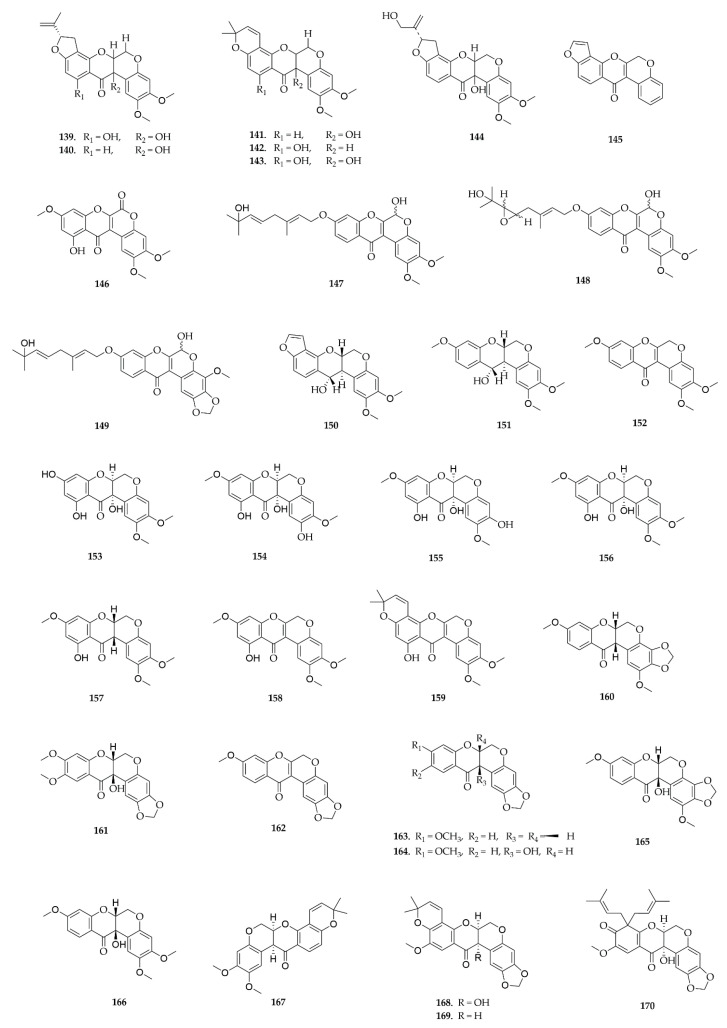
Rotenoids reported in this review.

Rotenoids are specialized metabolites found exclusively in species of the Papilionaceae subfamily (Figure 12), with a higher incidence in the genus *Millettia*, especially *M. brandisiana*, although they have also been described in *Pseudarthria*, *Clitoria*, and *Xeroderris*. Most of the reported compounds were isolated from leaves; in contrast, (±)-Villosinol (**139**), (–)-cis-12a-Hydroxyrotenone (**140**), and (–)-Tephrosin (**141**) from *M. brandisiana*, were obtained specifically from roots [46,47]. Less frequently, molecules isolated from *Millettia pinnata* seeds, such as Pongarotene (**145**), and from *Millettia caerulea* fruits, which provided compounds **168**–**170**, were also observed [48,49].

Organic solvents such as dichloromethane, ethyl acetate, ethanol, and methanol/dichloromethane mixtures are predominantly used for extraction of rotenoids, reflecting the relatively nonpolar nature of these compounds. For example, the oblarotenoids (**160**–**165**) isolated from *Millettia oblata* ssp. teitensis and (−)-Tephrosin (**141**), which were isolated from a 1:1 mixture of methanol/dichloromethane [50].

From the leaves of *M. brandisiana*, the use of ethyl acetate allowed the isolation of compounds **153**–**159** [39]. These rotenoids show structural similarity, differing mainly in the number and position of methoxyl groups, in addition to the occasional presence of prenylated substituents. This profile reinforces the efficiency of ethyl acetate in the extraction of metabolites of intermediate polarity, highlighting its ability not only to recover oxygenated derivatives, but also prenylated and methoxylated compounds.

From the ethanolic extract of *Xeroderris stuhlmannii* leaves, it was possible to isolate substances **147**–**149**, with a similar rotenoid nucleus but with different prenylated side chains, with varying patterns of oxygenation and unsaturation [51]. These structural differences reinforce the use of ethanol as a solvent of intermediate polarity, which would be the choice for extracting molecules of different polarity ranges.

## 5. Pharmacological Properties

### 5.1. Aurones

Only two activities were evaluated for this group of compounds: antioxidant and neuroprotective activities. The compound sulphuretin (**1**) showed antioxidant activity with an IC_50_ value of 25.3 ± 0.3 μM [52], higher than the positive control used (ascorbic acid, 45.6 ± 0.6 μM). Compounds **3** to **11** were evaluated for neuroprotective activities against 6-OHDA-induced cell death in SH-SY5Y cells with curcumin as the reference compound. The compounds altilisin (**3**), (*Z*)-4-*O*-*β*-*D*-glucopyranosyl-7,3′, 4′-trihydroxyaurone (**4**) and (*Z*)-3′-*O*-*β*-*D*-Glucopyranosyl-4,5,6,4′-tetrahydroxy-7,2′dimethoxyaurone (**5**) showed strong and moderate neuroprotective activity with IC_50_ values of 3.56, 6.02, and 4.72 μM, respectively. The other compounds were considered inactive as they showed higher IC_50_ than the curcumin (reference compound, IC_50_= 6.51 μM) [27].

### 5.2. Biflavonoids

Although biflavonoids have several related biological activities, such as antimicrobial, anti-osteoporotic, and antitumor activity [53,54], none of the reports on the occurrence of biflavonoids in Fabaceae species during the last decade, compiled in this study, had their pharmacological properties investigated. The evaluation of the pharmacological properties of this class of substances is limited by several possible factors. These molecules exhibit structural complexity, including regioisomerism and atropisomerism, presenting complex NMR and mass spectrometry characterization and restricting the definition of structure-activity relationships [55,56]. In addition, low extraction yields and synthetic challenges reduce the availability of pure standards for screening and mechanistic studies [55,56,57]. Added to this are pharmacokinetic limitations, such as low aqueous solubility, poor oral absorption, and extensive plasma protein binding. Although formulation approaches have demonstrated significant improvements in solubility and bioavailability, this additional requirement reinforces the barriers to advancing pharmacological investigations of this subclass of flavonoids [58,59].

### 5.3. Coumestans

Coumestans are a class of metabolites that show a variety of biological activities, including estrogenic, anti-cancer, anti-inflammatory, antimicrobial, anti-diabetic, neuroprotective, anti-obesity, anti-osteoporotic, immunosuppressive, and antioxidant activity [7]. In this report, the compounds from Fabaceae were evaluated for their antimicrobial activity against protozoa. Compounds **42**, **43,** and **44**, isolated from *Campylotropis hirtella*, underwent in vitro antimalarial activity evaluation, the IC_50_ values for coumestans ranged from 354.5 to 453.5 µM, which can be considered weak activity when compared to the standard drug (chloroquine diphosphate, IC_50_ = 69.5 µM) [23].

The coumestans were evaluated for their activity in metabolic disorders, such as inhibition of diacylglycerol acyltransferase (DGAT), an enzyme responsible for the synthesis of triglycerides (TGs), thus, preventing their synthesis can lead to improvement in obesity and other cardiovascular diseases. Molecules **27**–**29** were tested for inhibition of DGT1, Bavacoumestan C (**28**) showed an IC_50_ value of 52.3 ± 1.3 µM, the best result when compared to **27** and **29**, which showed values of 65.2 ± 1.1 and 116.5 ± 1.2 µM, respectively. Compound **28** also showed high inhibitory activity of α-glucosidase (31.2 µM) when compared to its inhibitor, acarbose (214.8 µM) [21]. Dalbergestan (**47**) was evaluated for insulin-secreting activity in isolated mouse islets (MIN6 cells), with arginine serving as the standard insulin secretagogue. The compound showed no significant effect on insulin secretion, producing only a 42.1% increase compared to the baseline, whereas arginine induced a 299.5% increase [29].

In addition to these activities, coumestrol (**25**) exhibited an inhibitory effect on osteoclastogenesis, which led to the evaluation of compounds glytabastan A-H (**30**–**37**) and dolichosin A (**38**) no RANK-induced RAW 264.7 macrophage model. Coumestan **38** showed the greatest inhibition of osteoclastogenesis at a concentration of 20 µM, as it showed the lowest release of p-nitrophenol, an indicator of cell differentiation. Next, compounds **30**, **31,** and **25** exhibited moderate anti-arthritic activity, while molecules **32**–**37** showed no inhibitory activity [38].

### 5.4. Homoisoflavonoids

The pharmacological exploration of homoisoflavonoids remains limited, as many compounds have been identified but lack systematic biological evaluation. However, several molecules have demonstrated relevant pharmacological potential. Cropalliflavone B (**49**), isolated from Crotalaria pallida, demonstrated moderate antiproliferative activity against the MCF-7 cell line, exhibiting an IC_50_ value of 6.77 mM when compared to the positive standard Adriamycin (IC_50_ 0.23 ± 0.018) used in the assay. These results show that despite having a higher IC_50_ than the positive control, this substance can be considered a promising anti-cancer agent, with further investigation into the mechanisms of action and structure-activity relationships being necessary in order to achieve greater potentiation of this inhibition of cell proliferation [32,43].

### 5.5. Neoflavonoids

The compounds Melanettin (**55**) and Melannein (**56**) were evaluated for cardioprotective activity in ischemia–reperfusion (I/R) injury in H9c2 cells. The cardioprotective effects evaluated in this study assessed the inhibition of myeloperoxidase (MPO) protein as a strong contributor to myocardial injury involved in cardiac dysfunction processes. However, the compounds did not show significant cardioprotective effects, as they exhibited EC_50_ values greater than 157.44 ± 3.61 μM [44].

### 5.6. Pterocarpans

#### 5.6.1. Antitumoral Activity

Among the pharmacological activities of this class of compounds, cytotoxicity activity is particularly important. A total of nine compounds exhibited cytotoxic activity against various tumor cell lines with IC_50_ values ranging from 5.99 to 39.66 µM. Most substances were tested against KB (human epidermoid carcinoma) and HeLa (human cervical carcinoma) cells (Table 1). The compound Velucarpin C exhibited the lowest IC_50_ values of 8.69 and 8.09 µM and 5.99 and 8.22 µM against the KB and HeLa cell lines, respectively, demonstrated more effective cytotoxicity when compared to the other compounds reported in this study, which exhibited IC_50_ values between 15.77 µM and 30.19 µM (HeLa).

The compounds Dehydromaackiain (Maackianin) (**66**), Flemichapparin B (**67**), and 3,9-Dihydroxypterocarp-6a-en (**69**) were evaluated for their cytotoxic activity against four human tumor cell lines in addition to HeLa: HepG2 (hepatocellular carcinoma), MCF-7 (breast carcinoma), HCT-116 (colorectal carcinoma), and MDA-MB-231 (triple-negative breast carcinoma). The IC_50_ values obtained revealed distinct activity profiles among the compounds. Dehydromaackiain (**66**) had the lowest IC_50_ values among the three, indicating greater cytotoxic potential, especially against the HepG2 line (13.39 ± 1.41 µM), followed by MCF-7 (21.21 ± 0.93 µM), HCT-116 (21.90 ± 1.73 µM), and MDA-MB-231 (25.45 ± 2.09 µM). These results suggest that these compounds exhibit diverse cytotoxic potential, with a more evident action on liver and breast cells. Flemichapparin B (**67**), in turn, showed moderate cytotoxicity in this report, with emphasis on the MCF-7 (21.10 ± 1.65 µM) and MDA-MB-231 (22.76 ± 3.54 µM) lines. The other values were 25.38 ± 1.92 µM for HepG2, 27.03 ± 1.64 µM for HCT-116, and 30.19 ± 0.54 µM for HeLa, indicating a less potent action profile compared to Dehydromaackiain (**66**), which exhibited IC_50_ values of 13.39 ± 1.41 µM for HepG2, 21.90 ± 1.73 µM for HCT-116, and 22.50 ± 1.09 µM for HeLa. The compound 3,9-dihydroxypterocarp-6a-en (**69**) presented the highest IC_50_ values, demonstrating the lowest cytotoxic potential among the compounds reported here, compared to MCF-7 (30.34 ± 1.32 µM), HepG2 (34.25 ± 1.87 µM), HeLa (36.15 ± 7.34 µM), MDA-MB-231 (36.78 ± 5.61 µM), and HCT-116 (39.66 ± 2.06 µM) [53].

Indigocarpan (**82**) exhibited antimetastatic efficacy by modulating matrix metalloproteinases and promoting cell death, with the following IC_50_ values for cytotoxicity in three cell lines: 92 μg/mL (breast cancer cell line MDA-MB-231), 93 μg/mL (prostate cancer cell line PC3), and 135 μg/mL (lung cancer cell line A549). The study also demonstrated a strong antimetastatic activity, inhibiting cell–cell contact, cell migration, and the expression and activity of matrix metalloproteinases (MMP2 and MMP9) in the MDA-MB-231 and PC3 cell lines [53]. Additionally, Erybraedin C (**87**) also showed a significant reduction in cell viability of neuroblastoma (SH-SY5Y) cells [54].

#### 5.6.2. Antimicrobial Activity

The pterocarpans were also investigated for their antimicrobial potential, including activity against bacteria, fungi, protozoa, and viruses. Among them, Vouacapan (**100**) exhibited notable antifungal activity, with a minimum inhibitory concentration (MIC) of 0.98 µg/mL against *Candida albicans* ATCC 10231, demonstrating potency that is comparable to or even greater than that of standard antifungal agents such as fluconazole and amphotericin B (MIC 1 and 4 µg/mL, respectively) [60].

In the context of antiparasitic activity, Melilotocarpan C (**116**) showed weak antiplasmodial effects, with an IC_50_ of 42 ± 5 µM against the drug-resistant *Plasmodium falciparum* Dd2 strain [59]. Furthermore, several pterocarpan derivatives—Erythrabyssin II (**66**), 1-Methoxyerythrabyssin II (**78**), Bicolosin A (**125**), Bicolosin B (**126**), and Lespebuergine G4 (**123**)—were found to inhibit the papain-like protease (PLpro) activity of SARS-CoV in a dose-dependent manner, with inhibition constants (K_i_) ranging from 5.56 to 75.37 µM, highlighting their potential as antiviral agents [62].

#### 5.6.3. Other Activities

Other biological activities observed for pterocarpans include anti-inflammatory activity, antivenom, hepatoprotective effects and enzymatic inhibitors. Sophoratonkin (**112**), which exhibited an inhibitory effect on NO production in LPS-stimulated RAW 264.7 cells with an IC_50_ of 33.0 µM [63]. Maackiain (**97**) was found to be an inhibitor of collagen-induced platelet aggregation (IC_50_ at 100 µM = 35.2 ± 14.4 and at 50 µM = 71.4 ± 4.3) [62], as well as having an inhibitory effect on nitric oxide production induced by LPS in RAW 264.7 cells (IC_50_ 27.0 µM) [63].

The compound Homopterocarpin (**106**) was evaluated for its potential to combat liver injury and oxidative stress in paracetamol-induced hepatotoxicity and was shown to significantly restore elevated levels of serum marker enzymes, thiobarbituric acid reactive substances (TBARS), GSH levels, and GPx activity to near-normal values. The compound also protected against the acetaminophen (APAP)-induced increase in ALT activity, being effective at lower doses, although it showed a possible pro-oxidant trend at higher doses. Furthermore, the hepatoprotective activity of compound **106** may occur through the neutralization of toxic compounds produced during the conversion of paracetamol into a highly toxic metabolite via the cytochrome P450 pathway [39].

Cabenegrin A-II (**92**), isolated from the roots of *Harpalyce brasiliana*, has demonstrated antivenom activity by reversing biochemical, hematological, and hypotensive alterations induced by *Bothrops jararacussu* venom in rats [48]. Previously, Nakagawa et al. reported the antivenom effects of Cabenegrin A-I (**91**) and A-II (**92**) against *Bothrops atrox venom* in beagle dog’s male [47].

### 5.7. Rotenoids

The bioactive properties of the rotenoids concentrated mainly on cytotoxic and antimicrobial activities, demonstrating an important pharmacological relevance for these compounds.

#### 5.7.1. Cytotoxic Activity

Dalbinol (**144**) isolated from stems of *M. pachyloba* showed significant cytotoxic activity at a concentration of 50 µM against HeLa (human cervical cancer) and MCF-7 (human breast cancer) cancer cells, reducing cell viability by more than 70% and 60%, respectively. The lowest IC_50_ values observed for Dalbinol were against HCT-116 (16.65 µM) and HeLa (19.89 µM) cells [64].

Compounds **139**–**141** from *M. brandisiana* roots were evaluated for their cytotoxic activity against HepG2 (human liver cancer, specifically hepatocellular carcinoma), A549 (human lung cancer), HuCCA-1 (cholangiocarcinoma, bile duct cancer), MOLT- 3 (human T-cell acute lymphoblastic leukemia) and HeLa. **139**–**141** exhibited significant cytotoxicity, especially against MOLT-3 cells, with IC_50_ values of 0.61 µM, 0.27 µM, and 19.15 µM, respectively. (±)-Villosinol (**139**) stands out, presenting the broadest profile, with low IC_50_ values in different cell lines, including HeLa (0.87 µM). (–)-cis-12a-Hydroxyrotenone (**140**) showed selectivity for MOLT-3 and (–)-Tephrosin (**141**) demonstrated greater effect on HepG-2, both with lower activity against other cells [53].

Molecules **142**–**143** and **153**–**159** from leaves of *M. brandisiana* were tested against colorectal cancer (SW480), lung cancer (A549), and leukemia (K562) cells using the MTT assay. Rotenoids **153**–**157** were not active in the cell lines evaluated, except for (–)-(6aS,12aS)-6-deoxyclitoriacetal (**156**), which exhibited an IC_50_ of 248.22 µM. On the other hand, (–)-α-toxicarol (**142**) demonstrated the highest activity among the isolated rotenoids, with IC_50_ values of 104.4 µM, 136.6 µM, and 67.47 µM against the A549, SW480, and K562 cell lines, respectively [48].

#### 5.7.2. Antimicrobial Activity

Stuhlmarotenoid C (**149**) was evaluated for its antifungal and antibacterial activity against the fungi *Candida albicans*, *Candida krusei*, *Candida parasilosis*, and *Cryptococcus neoformans* and the bacteria *Pseudomonas aeruginosa*, *Staphylococcus aureus*, *E. coli*, *Klessiella pneumonae*, *Shigella flexineri*, *Shigella dysenteria*, *Salmonella typhimurium*, *Salmonella typhi*, and *Salmonella enteritidis*. Compound **149** showed MIC values of 125 μg/mL, demonstrating weak activity against *Shigella flexineri* and *Shigella dysenteria* and a moderate effect against *Salmonella typhi* (MIC = 62.5 μg/mL); for other bacteria strains, it showed MIC values greater than 125 μg/mL. Stuhlmarotenoid C also had weak to moderate effects against fungi, with MIC of 62.5 μg/mL for *C. parasilosis* and 125 μg/mL for *C. albicans* and *C. neoformans* [49].

*M. pyrrhocarpa* provided the isolation of compounds **150**–**152**, which were tested against *Staphylococcus aureus*, *Enterobacter aerogenes*, *Enterotoxigenic E. coli* (ETEC), *Enteropathogenic E. coli* (EPEC), Salmonella typhimurium, *Shigella flexneri*, *Proteus mirabilis*, *Vibrio cholerae*, and anti-HIV-1 activity. The three substances tested inhibited the growth of the nine bacterial strains evaluated, all presenting MIC of 3 mg/mL and MBC > 3 mg/mL. 6aS,12aS,12S-elliptinol (**150**) and 6aS,12aS,12S-munduserol (**151**) showed antiviral activity, with EC_50_ values of 4.48 and 4.99 µM, respectively, in reducing syncytium formation in 1A2 cells. By inhibiting the HIV-mediated cell fusion process, these molecules demonstrate potential as anti-HIV agents [65].

Compounds **141**, **160**–**168** isolated from *Millettia oblata* ssp. *teitensis* leaves were evaluated for their activity against respiratory syncytial virus (anti-RSV). Among the nine rotenoids tested, (–)-Tephrosin (**141**), Oblarotenoid A (**164**), 12a-hydroxymunduseron (**166**), and deguelin (**167**) showed the most significant activity, with IC_50_ values of 0.8 μM, 1.4 μM, 1.5 μM, and 0.4 μM, respectively, and a selectivity index above 10, indicating specific antiviral activity [40].

Pongarotene (**145**) isolated from *Millettia pinnata* seeds was tested for larvicidal activity against third-instar larvae of *Culex pipiens pallens*, *Aedes aegypti*, and *Aedes albopictus*. The molecule showed a lethal concentration (LC50) of 25.52 mg/L for *C. pipiens pallens*, 37.61 mg/L for *A. aegypti*, and 56.14 mg/L for *A. albopictus* [51].

## 6. LC-MS/MS Analysis of Rare Flavonoid Types from Fabaceae

Hyphenated analytical techniques, such as combining the efficient separation of HPLC with the structural information generated by NMR and mass spectrometry (MS), have been used to increase the speed and selectivity of phytochemical analysis of plant extracts [51]. Flavonoids are one of the classes of natural products most analyzed by LC-MS. However, there have been few studies on the use of this technique to search for rare flavonoids in plant species. The optimization of chromatographic separation conditions, as well as the choice of analytical technique, ionization source and mode and detectors, are determining factors for interpreting the structural information obtained by LC-MS analysis. Table 2 shows the LC-MS^n^ methods used in recent years for qualitative and quantitative analysis of compounds from the pterocarpan, neoflavonoid, and coumestan classes.

Most of the analyses were performed using HPLC or UPLC. Electrospray ionization (ESI) was the most widely used when compared to other ionization sources for the analysis of these compounds. Both positive and negative ionization modes were used for these analytes of interest. Studies show that in the negative ionization mode in ESI, the results found have a high sensitivity for low-acid polyphenols and aid in structural determination due to a wide variety of fragmentation reactions [66]. However, some characteristic fragmentation pathways, necessary for structural elucidation, are only detectable in positive ionization mode, for example, retro-Diels-Alder (RDA) fragmentation, which makes it possible to differentiate the classic skeletons of isoflavonoid derivatives such as pterocarpans. On the other hand, a variety of MS analyzers can be used to meet the objectives of each experimental design, such as QTOF, Ion Trap and Orbitrap. PDA is indispensable for aiding the structural identification of these compounds in these complex matrices.

**Table 2:** Liquid chromatography with Tandem Mass Spectroscopy method for rare analysis of flavonoid-types in different plants from Fabaceae.

Sample	Flavonoid-Type Rare Analyzed	Analytical Technique	Ionization Mode	Instrument Condition	Detector Conditions	Remarks	Ref.
Heartwood of *Dalbergia odorifera*	Neoflavonoids	UPLC-ESI-MS	[M − H] ^−^	C18 (2.1 mm × 100 mm, 1.8 μm) Mobile phase: 0.05% acetic acid in water; 0.05% acetic acid in CAN	UV and Orbitrap MS	A total of 13 compounds were classified as the skeleton of neoflavones with target analysis. Neutral losses of CH_3_, CO, H_2_O and CO_2_ are easily found in the skeletons of neoflavonoids.	[49]
Heartwook of *Pterocarpus santalinus*	Coumestans	UPLC- HRESI-MS-	[M + H] ^+^	C18 (2.1 mm × 150 mm, 1.7 μm) Mobile phase: water + 0.1% formic acid; ACN + 0.1% formic acid	UV and QTOF	The method allowed the guided isolation of the compound 3,9-O-dimethylcoumestrol.	[67]
Roots of *Glycyrrhiza inflata*, *G. uralensis* and *G. glabra*	Coumestans	HPLC-ESI-MS	[M + H] ^+^	C18 (4.6 mm × 250 mm, 5 μm) Mobile phase: water acidified with 0.2% *v*/*v* formic acid; acetonitrile	PDA and TripleTOF	A total of 39 compounds were identified, including two coumestans, glycycoumarin and neoglycyrol.	[68]
Root of *Ononis spinosa* L.	Pterocarpans	LC-ESI-MS	[M + H] ^+^	C18 (3.0 mm × 150 mm, 3.5 μm) Mobile phase: 0.3% (*v*/*v*) formic acid and methanol	PDA, QTOF and Ion Trap	The method made it possible to differentiate fragmentation patterns of isomeric forms of Maackiain and described the fragmentation pattern of Medicarpin for the first time.	[69]
Roots of *Milletia speciosa*	Pterocarpans	UPLC-ESI-MS	[M − H] ^−^	C18 (2.1 mm × 100 mm, 1.7 μm) Mobile phase: water acidified with 0.1% *v*/*v* formic acid and ACN	PDA and QTOF	A total of 38 components, including Maackiain pterocarpan were unambiguously identified or tentatively assigned.	[70]
Stems of *Acosmium diffusissimum*	Pterocarpans	LC-ESI-MS	[M + H] ^+^	C18 (4.6 mm × 250 mm 5 μm) Mobile phase: water acidified with 0.1% *v*/*v* formic acid and methanol	PDA and Ion Trap	The method enabled the isolation of new pterocarpans through the analysis of fragmentation patterns and the molecular network approach.	[66]

**Figure 13:**
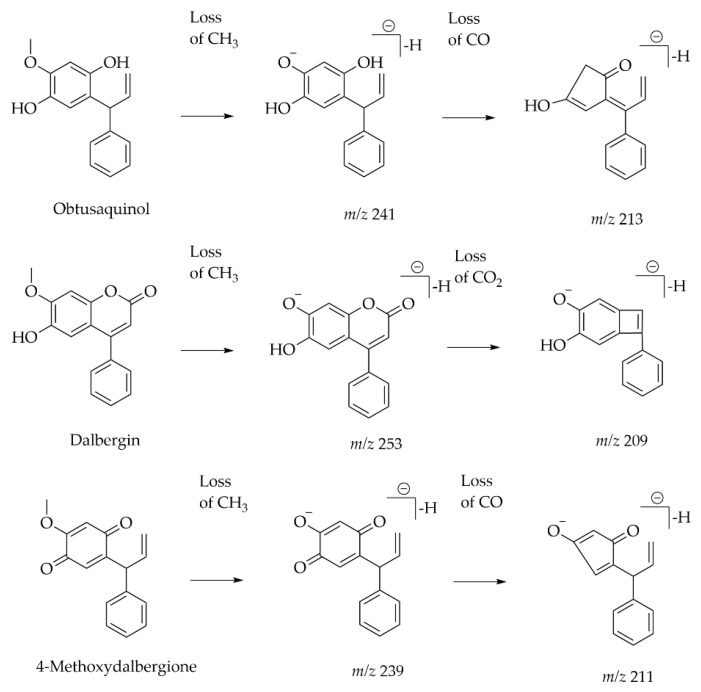
Fragmentation patterns and characteristic fragments in negative mode of neoflavonoids [49].

A total of 140 flavonoids from the heartwood of *Dalbergia odorifera* were tentatively characterized using RP-UPLC with ESI-MS, in which 13 compounds were classified as skeleton neoflavones with marked analysis: 3′-Hydroxymelanettin, Melannein, 2-(2′-Methoxy,4′-Hydroxy)-Aryl-3-Methyl-6-Hydroxy-Benzofuran, Pterolinus B, Melanettin, Dalbergin, Gnaphaliin. 3′-O-Methylviolanone, Melannein Isomer, Obtusafuran, Pterostilbene [49]. This study showed that neutral losses of CH_3_, CO, H_2_O, and CO_2_ fragments occur easily in neoflavonoid skeletons (Figure 13).

Fang et al. (2016) identified a total of 39 compounds and observed that the RDA fragmentation that generates the ^1,3^A^+^ and ^1,3^B^+^ fragments commonly observed for flavones, isoflavonones, and chalcones is not observed for coumestans [68]. Also in this study, a neutral loss of C_5_H_11_ was observed for the compounds neoglycyrol and glycycoumarin, adding to the known possibilities of fragmentation patterns involving losses of CO, C_4_H_8_ and C_5_H_8_O, characteristic of prenylated coumestans. The compound 3,9-*O*-dimethylcoumestrol exhibiting an ion of *m*/*z* 297.0758 [M + H]^+^ was isolated, guided by the LC-MS/MS analyses developed by Wasilewicz et al. (2023) in their first report about using the molecular networking for this class of compounds [67].

**Figure 14:**
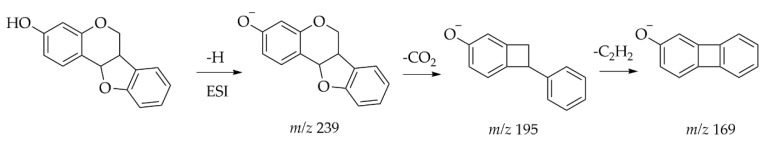
Fragmentation pattern of 3-hydroxypterocarpan in negative mode by ESI-MS. Adapted from Goel et al., 2013 [71].

**Figure 15:**
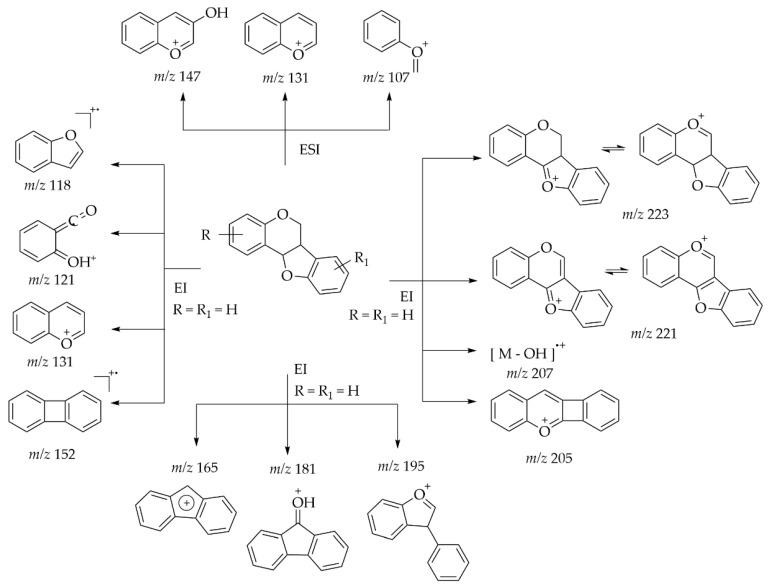
Fragmentation pattern of pterocarpans in the positive mode by EI-MS and ESI-MS. Adapted from Goel et al., 2013 [71].

On the other hand, pterocarpans belong to the class of rare flavonoids most studied using the LC-MS/MS technique. Goel and collaborators (2013) compiled a report on the synthesis, stereochemistry, structural classification, and chemical reactivity of naturally obtained pterocarpanes, including important characteristics that allow the structural identification of these compounds using MS, data obtained by electron impact or electrospray ionization, in both negative and positive modes (Figure 14 and Figure 15) [71].

**Figure 16:**
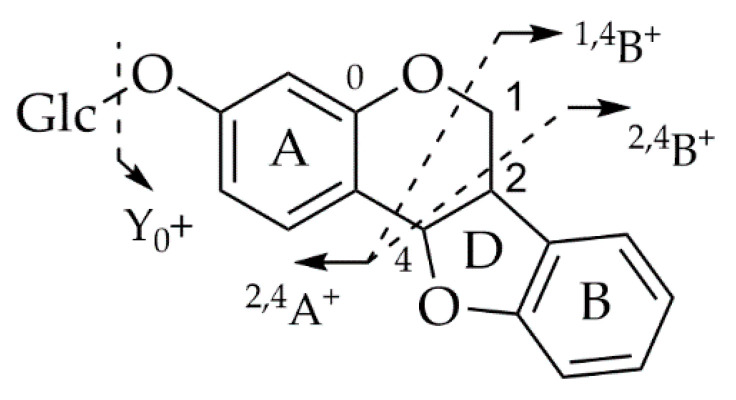
General fragmentation mechanism for pterocarpans from roots of *Ononis spinosa*. Adapted from Gampe et al., 2016 [69].

This fragmentation proposal, which is well-established in the literature, also led to the identification of the compound Maackiain by Gampe et al. (2016) and four other compounds in which it was possible to deduce a general fragmentation mechanism for pterocarpans present in the roots of *Ononis spinosa* (Figure 16) [69]. The identification of Maackiain in the roots of *Milletia speciosa* was also possible due to the reports previously obtained from the studies by Yu and collaborators (2019) [70]. It also allowed Sousa et al. (2025) to use a molecular networking approach for LC-MS/MS-guided isolation of new pterocarpans and proposed a fragmentation route for the compounds diffusicarpan A and diffusicarpan B from the stems of *Acosmium diffusissimum* [66].

Although reports on the use of LC-MS/MS to characterize biflavonoids in species from the Fabaceae family are rare, this class of compounds has been extensively studied for species from other families such as Thymelaeaceae and Selaginellaceae. Generally, studies on reported fragmentation patterns use LC-ESI-MS (+), but negative mode ionization also proves to be sensitive and selective for analysis of these compounds. Yao et al. (2017) identified the isoflavonoids of *S. doederleinii* using HPLC-QTOF MS/MS in (-) ESI mode and proposed fragmentation routes for the four most frequent categories in the species, the biflavonoids IC3′-IIC8′, IC3′-IIC6′’, IC3′-IIC3′’ and C-O linked, thus demonstrating that the type of linkage has significant impacts on the identification pattern of these compounds in complex matrices [72].

**Figure 17:**
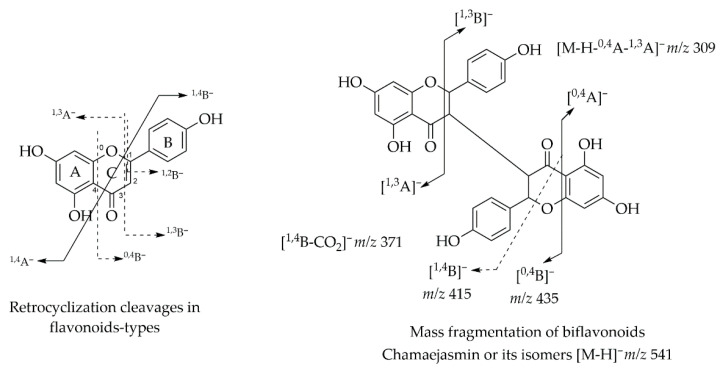
Retrocyclization cleavage in flavonoids-types and mass fragmentation of biflavonoids IC3-IIC3 linked (Chamaejasmin or its isomers). Adapted from Ko et al., 2013 [73].

In this report, Fabaceae species mostly produce biflavonoids of the IC3-IIC3 linked type, such as chamaejasmine derivatives. In this type of linked flavonoid compounds, the product ions generated are characteristic of RDA fragmentations, as expected for classical flavonoids. Studies also propose that biflavonoids can exhibit the following fragmentation patterns, depending on the flavonoid subtype attached: ^1,3^B^−^, ^1,3^A^−^, e ^1,4^B^−^ + 2H in flavones; ^1,2^A^−^, ^1,2^B^−^ e^1,3^B^−^ in flavonols; and e ^1,3^A^−^, ^1,3^B^−^ e ^1,4^A^−^ in flavonones. (Figure 16) The compounds chamaejasmin and its isomers neochamaejasmin and isochamaejasmin present the base peak of the product ion in MS/MS with *m*/*z* 415 indicating the neutral loss of 126 Da, indicating the loss of the C_6_H_6_O_3_ portion generated from a ^1,4^B^−^ fragmentation. Other characteristic ions can be generated from the retrocyclization reactions shown in Figure 17. Fragment ions generated from common neutral losses of CO and CO_2_ can also be found due to the C ring of the flavonoid-type skeleton, as well as neutral losses of H_2_O and 15 Da associated with the presence of hydroxyl or methoxyl groups in the structures [73].

In light of this, LC-MS studies play a crucial role in the investigation of other secondary metabolite classes that are both rare and promising. Such analyses, through targeted and untargeted approaches, enable not only the identification of novel, unprecedented compounds of chemical interest but also the assessment of their pharmacological potential. In this context, the application of LC-MS significantly contributes to expanding the chemical knowledge of these classes and uncovering new perspectives for the development of bioactive agents.

## 7. ADMET Analysis

The compounds herein demonstrate a wide range of pharmacological activities, such as antitumor, antiviral, antimicrobial, and anti-inflammatory, among others. Like other classes of natural products, their unique chemical features and generally favorable pharmacokinetic profiles make them attractive for drug discovery and development [74]. Meanwhile, drug development is a complex and expensive process involving several stages, ranging from target selection, discovery of active compounds and optimization to clinical trials. Nevertheless, many candidates fail, mainly due to low efficacy or safety issues. These failures highlight the importance of absorption, distribution, metabolism, excretion, and toxicity (ADMET) in all stages of development. Based on that, efficiency, potency, and ADMET properties are now evaluated early, allowing the elimination of unsuitable compounds before costly steps [75,76].

The increasing availability of experimental ADMET data has enabled the development of numerous in silico models to support early-stage drug development, such as SwissADME, ADMETlab, and vNN-ADMET, among others [77]. By integrating kinetic data into comprehensive predictive frameworks, these tools help to anticipate the in vivo behavior of drug candidates. When applied in combination with in vitro and in vivo methods, computational ADMET models reduce safety risks, lower failure rates, and minimize the use of animals [75,78,79].

Drug-likeness is closely related to ADMET properties. While ADMET directly describes the pharmacokinetic and toxicological behavior of molecules, drug-likeness refers to structural and physicochemical features commonly that are found in approved drugs or promising candidates [80]. In this sense, a widely used approach involves property-based filters that define acceptable ranges for molecular features such as lipophilicity (logP), molecular weight, the number of hydrogen bond donors and acceptors. Lipinski’s Rule of Five is the most well-known of these, setting criteria for oral bioavailability [81]. Additional filters, such as the Ghose, Veber, and Egan rules expanded on these parameters to better capture the relationship between molecular properties and drug behavior [82]. Altogether, these tools help streamline compound selection and reduce the likelihood of failure in later stages of drug development by helping to eliminate compounds with poor ADMET profiles in pharmaceutical research.

**Figure 18:**
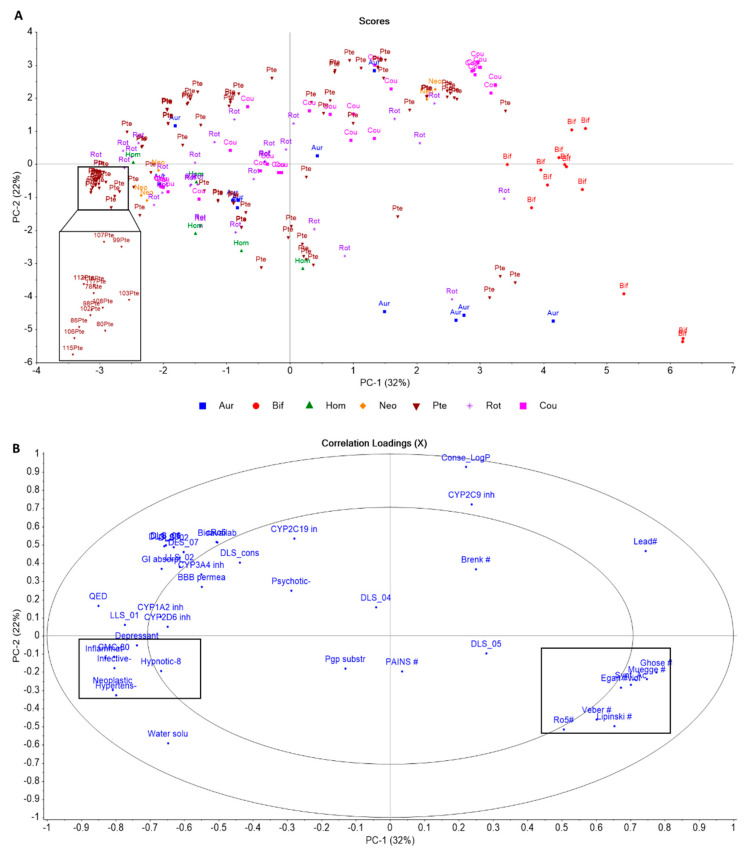
PCA score plot of Fabaceae flavonoids (**A**). Weight plot (**B**) demonstrates the qualitative ADMET and Drug-likeness descriptors used to perform classification. Highlights in red indicate the best (right side of the figure) and worst profiles (left side of the figure).

Considering the bioactivity of uncommon flavonoids obtained from Fabaceae family and their potential for drug development, we have established a small dataset including the 170 unique compounds considered in this review and estimated their ADMET and drug-likeness profiles. In order to identify chemical patterns across the seven subclasses, the results were represented by a PCA (Principal Component Analysis) plot. The compounds were ordered according to their scaffolds, indicated by colors and labels containing the initial letters of each group, Figure 18.

The analysis of the score graph in Figure 18A reveals a total variance of 54% and clearly distinguishing biflavonoids (colored in red and encoded as “Bif”) from the other compounds. The loading plot (Figure 18B) summarizes the 42 selected drug-likenes and ADMET parameters. The correlation demonstrated in the plots suggests that as long as data distribution tends to the quadrants 2 and 4, their ADMET and Drug-likeness become unfavorable. Hence, among the seven subclasses the biflavonoids, some pterocarpans and aurones fall outside the optimal range of drug-like compounds, as predicted by different algorithms. These include the Rule of Five (R05), Egan’s rule, Ghose’s rule, Veber’s rule, Muegge’s rule, alongside their noted violations (represented by #, highlighted in dark, Figure 18B). Additionally, their potential to act as inhibitors of many Cytochrome P450 enzymes (represented by “CYP inh”) is also indicated. A clear correlation between the variable “Conse_LogP” (which represents the median of lipophilicity estimated by five different models) and “CYP2C9 inh” suggests that the higher the lipophilicity, the greater are the chances for these compounds inhibiting this CYP isoform.

On the other hand, most part of the compounds sorted in quadrants 1 and 3 (Figure 18A), exhibit favorable profile as observed by the presence of key descriptors, Figure 18B. The quantitative estimate of drug-likeness (QED) reflects the underlying distribution of molecular properties establishing a numerical scale ranging from zero (all properties unfavorable) to one (all properties favorable), representing a sensible way to measure dug-likeness potential, surpassing the classical limitations of binary classification. Drugs with high QED scores exhibit higher absorption and bioavailability, are administered at lower doses, and have fewer drug–drug interaction warnings, P-glycoprotein interactions, and absorption issues due to a food effect [83,84].

Another key descriptor is “Water solu”, it favors grouping together the water-soluble compounds, an import feature to the development of drugs. Additionally, also grouped in quadrant 3 (Figure 18B, highlighted in black), there are the sets based on approved drugs, such as anti-inflammatory, antidepressant, anti-infective, and antineoplastic. The encoded descriptors encompass the compounds that share at least 80% of physicochemical properties similar with those approved drugs, indicating a high potential to discover and develop new medicines, related to the referred activities. Altogether, the analysis of score and loading plots allowed the identification of some promising compounds, among them a small group of pterocarpans (highlighted in black, Figure 18A) including thirteen compounds stands out. Among them, only three were tested and demonstrated biological activity; melilotocarpan C (**116**), maackiain (**97**), and homopterocarpin (**106**). These findings demonstrated the pharmacological potential of these uncommon flavonoids, and at the same time drew attention to the fact that only 40% of the 170 compounds had been tested, demonstrating a huge chemical space uncovered in biological studies.

## 8. Conclusions

This review highlights the occurrence of uncommon flavonoids distributed in seven classes of compounds aurones, biflavonoids, coumestans, homoisoflavonoids, neoflavonoids, pterocarpans, and rotenoids that are frequently found in the Fabaceae family, specifically in the Papilionoideae and Caesalpinioideae subfamilies, enriching the chemical diversity of these species. The structural diversity observed in the 170 compounds compiled in this review supports the existence of a highly specialized biosynthesis that still requires more advanced studies in order to elucidate biosynthetic routes which are still poorly explored. The presence of these compounds mostly in the roots suggests that this tissue is one of the main ones responsible for gathering the enzymatic arsenal for producing these compounds. The LC–MS analysis data reported here for these compounds can inform chemotaxonomic and metabolomic studies assisted by Global Natural Products Social Networking (GNPS) or other dereplication techniques and can also serve as a basis for investigating new compounds of these rare flavonoid types with bioactive potential.

The pharmacological data reported demonstrates the potential of these compounds for a wide variety of biological properties including antimicrobial activity, antioxidant and anti-inflammatory activity, as well as cardio- and hepatoprotective effects. Undeniably, cytotoxic activity deserves to be highlighted since it has been observed for compounds from various classes, especially rotenoids and pterocarpans, the latter of which has been widely studied for its effects against numerous cell lines. Given these data, this work also demonstrates the potential of these compounds for the development of new drugs, with reference to the physicochemical properties that favor the pharmacokinetic parameters associated with good oral availability.

Among the subclasses compiled, several phytomolecules stand out for their promising pharmacological profiles. In the aurone group, altilisin exhibited strong neuroprotective activity (IC_50_ = 3.56 µM), while sulphuretin showed potent antioxidant effects (IC_50_ = 25.3 µM). Among the coumestans, bavacoumestan C demonstrated significant inhibition of DGAT1 (IC_50_ = 52.3 µM) and α-glucosidase (31.2 µM), and dolichosin A showed notable osteoclastogenesis inhibition at 20 µM. In the pterocarpan class, velucarpin C (IC_50_ ≈ 8 µM), maackiain (HepG2 IC_50_ ≈ 13.4 µM), and erybraedin C (0.1976 µg mL^−1^ in SH-SY5Y) exhibited marked cytotoxicity, while vouacapan presented potent antifungal activity (MIC 0.98 µg mL^−1^). For the rotenoids, deguelin and (−)-tephrosin displayed strong antiviral activity (IC_50_ ≈ 0.4–0.8 µM), and (±)-villosinol showed submicromolar cytotoxicity against selected tumor cell lines. In contrast, biflavonoids and neoflavonoids remain less explored pharmacologically. Altogether, these examples reinforce the therapeutic promise of rare Fabaceae flavonoids and underscore the need for standardized in vitro and in vivo evaluations as well as further ADMET investigations to advance their preclinical development.

In general, the chemical diversity favored the ADMET profile evaluated, except for the biflavonoids. These findings reinforce the chemical and pharmacological potential of this class of compounds, highlight the chemical richness of plants in the Fabaceae family and guide future research into new compounds, the development of chemotaxonomic studies, and technological innovation aimed at discovering new drugs.

## Figures and Tables

**Table 1:** Compounds belonging to the pterocarpan class with cytotoxic activity against selected cell lines.

Compound	Cell Line	IC_50_	Ref.
Indigocarpan (**82**)	MDA-MB-231, PC3, A549	92 μg/mL, 93 μg/mL, 135 μg/mL	[53]
Erybraedin C (**87**)	SH-SY5Y	0.1976 µg/mL	[54]
Dehydromaackiain (**66**)	HeLa, HepG2, MCF-7, HCT-116, MDA-MB-231	22.50 ± 1.09 µM, 13.39 ± 1.41 µM, 21.21 ± 0.93 µM, 21.90 ± 1.73 µM, 25.45 ± 2.09 µM	[60]
Flemichapparin B (**67**)		30.19 ± 0.54 µM, 25.38 ± 1.92 µM, 21.10 ± 1.65 µM, 27.03 ± 1.64 µM, 22.76 ± 3.54 µM	
3,9-dihydroxypterocarp-6a-en (**69**)		36.15 ± 7.34 µM, 34.25 ± 1.87 µM, 30.34 ± 1.32 µM, 39.66 ± 2.06 µM, 36.78 ± 5.61 µM	
Velucarpin A (**119**)	KB, HeLa	15.77 µM, 18.96 µM	[61]
Velucarpin B (**120**)		19.96 µM, 25.24 µM	
Velucarpin C (**121**)		8.22 µM, 8.09 µM	

## Data Availability

All data generated or analyzed during this study are included in this article and its Supplementary Material.

## Supplementary Materials

The following supporting information can be downloaded at: https://www.mdpi.com/article/10.3390/plants14233549/s1, Table S1: Aurones; Table S2: Biflavonoids; Table S3: Coumestans; Table S4: Homoisoflavonoids; Table S5: Neoflavonoids; Table S6: Pterocarpans; Table S7: Rotenoids; Refs. [9,27,28,31,32,33,44,45,48,52,53,54,60,62,63,78,80,85,86,87,88,89,90,91,92,93,94,95,96,97,98,99,100,101,102,103,104,105,106,107,108,109,110,111,112,113,114,115,116,117,118,119,120,121] has cited in Supplementary materials.
