# Supplementary material for: Fabaceae Flavonoids Beyond the Commonplace: A Review of Chemical Diversity, Pharmacological Activities, Mass Spectrometric Profiling and In Silico Insights into Their Subclasses
Source: Plants (Basel). 2025 Nov 21;14(23):3549. doi: 10.3390/plants14233549 (PMC12694499; doi:10.3390/plants14233549)
Supplement: Supplementary file 1 [file plants-14-03549-s001.zip › plants-3945860-supplementary.pdf]

plants

SUPPLEMENTARY MATERIAL

Review

Fabaceae Flavonoids Beyond the Commonplace: A Review of Chemical Diversity, Pharmacological Activities, Mass Spectrometric Profiling and *In Silico* Insights Into Their Subclasses

Ana Rita Rodrigues de Almeida Silva Brilhante [1,†], Gabriela Ribeiro de Sousa [1,†], Ana Karoline Silva de Aquino-Vital [1], Natanael Teles Ramos de Lima [1], Ranna Beatris de Lima Souza [1], Thalisson Amorim de Souza [1], Marcus Tullius Scotti [1,2], José Maria Barbosa Filho [1,3], Josean Fechine Tavares [1,3,*] and Marcelo Sobral da Silva [1,3].

[1] Graduate Program in Natural and Synthetic Bioactive Products, Federal University of Paraiba, João Pessoa 58051-900, Brazil; thalisson.amorim@ltf.ufpb.br (T.A.d.S.); mtscotti@gmail.com (M.T.S.); barbosa.ufpb@gmail.com (J.M.B.F.); marcelosobral@ltf.ufpb.br (M.S.d.S.)

[2] Department of Chemistry, Exact and Natural Sciences Centre, Federal University of Paraiba, João Pessoa 58051-900, Brazil

[3] Department of Pharmaceutical Sciences, Health Sciences Centre, Federal University of Paraiba, João Pessoa 58051-900, Brazil

* Correspondence: josean@ltf.ufpb.br

† These authors contributed equally to this work.

CONTENT

Table S1. Aurones 4

Table S2. Biflavonoids 5

Table S3. Coumestans 7

Table S4. Homoisoflavonoids 9

Table S5. Neoflavonoids 10

Table S6. Pterocarpans 11

Table S7. Rotenoids 16

Table S1. Aurones

N°	Compound	Species	Plant part	Extract solvent	Activity	Reference
**01**	Sulfuretin	*Cassia nomame*	Aerial parts	Methanol	Antioxidant	[51]
**02**	Astrernestin	*Astragalus ernestii*	Roots	Methanol	---	[28]
**03**	Altilisin I	*Sophora japonica*	Aerial parts	Ethanol	Neuroprotective	[27]
**04**	(*Z*)-4-*O*-*β*-*D*-glucopyranosyl-7,3′,4′-trihydroxyaurone					
**05**	(*Z*)-3′-*O*-*β*-*D*-Glucopyranosyl-4,5,6,4′-tetrahydroxy-7,2′dimethoxyaurone					
**06**	(*Z*)-4,5-methylenedioxy-6-hydroxybenzene-2[(2′,3′,4′-trimethoxy)-2-methyl-2(4-methyl-3-penten-1-yl)-2H-1-benzopyran-5-yl) methylene]-3-benzofuranon	*Sophora japonica*	Aerial parts	Ethanol	---	[27]
**07**	Cephalocerone					
**08**	(*Z*)-6,7,3′,4′-Tetrahydroxyaurone					
**09**	(*Z*)-4,6,4′-Trihydroxyaurone					
**10**	(*Z*)-4-Methoxy-6,4′-dihydroxyaurone					
**11**	(*Z*)-6-*O*-*β*-*D*-Glucopyranosyl-7,3′,4′-trihydroxyaurone					

Table S2. Biflavonoids

N°	Compound	Species	Plant part	Extract solvent	Activity	Reference
**12**	(+)-Liquiritigeninyl-(I-3, II-3)-naringenin	*Ormocarpum sennoides* subsp. *zanzibaricum*	Roots	Methanol (80 %)	---	[32]
**13**	Apigeninyl-(I-3, II-3) -naringenin	*Ormocarpum kirkii*	Bark	Ethanol	---	[33]
**14**	7-*O*-*β*-*D*-glucopyranosylchamaejasmin	*Ormocarpum kirkii*	Bark	Ethanol	---	[33]
**15**	Chamaejasmin	*Ormocarpum kirkii*	Bark	Ethanol	---	[33]
		Ormocarpum sennoides subsp. *zanzibaricum*	Roots	Methanol (80 %)	---	[32]
**16**	Ormocarpin	*Ormocarpum kirkii*	Bark	Ethanol	---	[33]
**17**	(-) - 7,7′′-di-*O*- *β*-*D*-glucosyl-(-)-chamaejasmin	*Ormocarpum kirkii*	Bark	Ethanol	---	[33]
**18**	(+) -5-hydroxy-7,4'-dimethoxyflavone-3*α*-2'''-hydroxy-4''',4''-dimethoxydihydrochalcone	*Poincianella pyramidalis*	Roots bark	Methanol	---	[84]
**19**	(+) -5,7-dihydroxy-4'-methoxyflavone-3*α*-2'''-hydroxy-4''',4''-dimethoxydihydrochalcone					
**20**	(-) -7-hydroxy-4'-methoxyflavone-3*α*-2''',4'''-dihydroxy-4''-methoxydihydrochalcone					

**21**	(-) -7,4'-dihydroxy-flavanone-3,8-5'',6'',4''-trihydroxy-flavone					
**22**	Rhuschalcone VI					
**23**	7,7'' -di-*O*-*β*-*D*-glucosylliquiritigeninyl-(I-3, II-3) – naringenin	*Ormocarpum kirkii*	Bark	Ethanol	---	[33]
**24**	trime-chamaejasmin	*Ormocarpum sennoides* subsp. *zanzibaricum*	Roots	Methanol (80 %)	---	[32]

Table S3. Coumestans

Nº	Compound	Species	Plant part	Extract solvent	Activity	Reference
**25**	Coumestrol	*Glycine tabacina*	Whole plant	Ethanol 95%	Antiarthritic	[85]
**26**	Sigmoidin K	*Erythrina variegata*	Roots	Chloroform	---	[86]
**27**	Bavacoumestan D	*Psoralea corylifolia*	Seeds	Ethanol 95%	DGAT inhibitory activity (obesity)	[87]
**28**	Bavacoumestan C					
**29**	Bavacoumestan B					
**30**	Glytabastan A	*Glycine tabacina*	Whole plant	Ethanol 95%	Antiarthritic	[85]
**31**	Glytabastan B					
**32**	Glytabastan C					
**33**	Glytabastan D					
**34**	Glytabastan E					
**35**	Glytabastan F					
**36**	Glytabastan G					
**37**	Glytabastan H					
**38**	Dolichosin A					
**39**	3,9-dihydroxy-10-methoxycoumestan	*Pongamia pinnata*	Roots	Ethanol 95%	Anti-inflammatory	[88]
**40**	3,9-dihydroxy-4-methoxy-benzo[4, 5]furo[3,2-c]chromen-6-one				---	
**41**	3'-methoxycoumestrol					
**42**	Campylohirtin A	*Campylotropis hirtella*	Roots	Ethanol 95%	Antimalarial	[89]

**43**	7,4′,5′-trihydroxy-5-methoxy-2-[3-methyl-2-butenyl]-coumestan	*Campylotropis hirtella*	Roots	Ethanol 95%	Antimalarial	[89]
					Antibacterial	[90]
**44**	Hirtellanine B	*Campylotropis hirtella*	Roots	Ethanol 95%	Antimalarial	[89], [90]
**45**	1-methoxylespeflorin I_2	*Lespedeza bicolor*	Roots	Methanol	Cytotoxicity	[91]
**46**	Lesbicoumestan					
**47**	Dalbergestan	*Dalbergia boehmii*	Leaves and hearthwood	Dichoromethane: methanol 1:1	Insulin Secretion	[92]

Table S4. Homoisoflavonoids

N°	Compound	Species	Plant part	Extract solvent	Activity	Reference
**48**	Cropalliflavone A	*Crotalaria pallida*	Seeds	Ethanol:Water (75:25)	---	[93]
**49**	Cropalliflavone B				Antiproliferative	
**50**	Metasappanin	*Caesalpinia bahamensis*	Stems	Ethanol 80%	---	[94]
**51**	Hematoxylol	*Haematoxylon campechianum*	Heartwood	Methanol	---	[111]
**52**	4-*O*-Methylhematoxylol					
**53**	Hematoxin					

Table S5. Neoflavonoids

N°	Compound	Species	Plant part	Extract solvent	Activity	Reference
**54**	3′-Hydroxymelanettin	*Dalbergia melanoxylon*	Heartwood	Ethanol	Cardioprotective	[44]
**55**	Melanettin					
**56**	Melannein					
**57**	Sophoraneoflavonoid A	*Sophora flavescens*	Roots	Ethanol	---	[45]
**58**	Sophoraneoflavonoid B					

Table S6. Pterocarpans

N°	Compound	Species	Plant part	Extract solvent	Activity	Reference
**59**	Eryvarin D	*Erythrina variegata*	Root bark	Acetone	---	[95]
		Erythrina lysistemon		Dichloromethane		[96]
**60**	Orientanol A	*Erythrina fusca*	Leaves	Methanol		[97]
**61**	Neorautenol	*Erythrina fusca*	Leaves	Methanol		[97]
**62**	2-(γ,γ-Dimethylallyl)-6a-hydroxyphaseollidin	*Erythrina brucei*	Root bark	Hexane	---	[98]
**63**	Phaseollidin	*Erythrina stricta*	Stem bark	Ethanol 95%		[99]
		Erythrina lysistemon	Root bark	Dichloromethane		[96]
		Erythrina subumbrans	Twigs and leaves	Ethanol 95%	---	[100]
**64**	Cristacarpin (Erythrabyssin I)	*Erythrina subumbrans*	Twigs and leaves	Ethanol 95%		[100]
		Erythrina fusca	Leaves	Methanol	---	[97]
**65**	Erythrabyssin II	*Erythrina lysistemon*	Root bark	Dichloromethane		[96]
		Lespedeza bicolor	Root bark	Methanol	Anti-coronaviral	[63]
**66**	Dehydromaackiain (Maackianin)	*Millettia pachyloba*	Stems	Ethanol:Water	Cytotoxic	[60]
**67**	Flemichapparin B	*Millettia pachyloba*	Stems	Ethanol:Water	Cytotoxic	[60]
**68**	Erycristagallin	*Millettia extensa*	Leaves	EtOAc	----	[101]
**69**	3,9-dihydroxypterocarp-6a-en	*Millettia pachyloba*	Stems	Ethanol:Water	Cytotoxic	[60]
**70**	Maackiain 3-O-β-D-glucoside-6''-O-piperidin-2-ylacetate	*Ononis spinosa*	Roots	Methanol:Water	---	[102]
**71**	(-)-trifolirhizin-6'-*O*-malonate	*Ononis angustissima*	Roots	Methanol:Water	---	[103]

**72**	Edunol	*Lonchocarpus bussei*	Roots	Dichloromethane: Methanol	---	[104]
		Harpalyce brasiliana	Heartwood	Ethanol	---	[48]
**73**	Calopocarpin	*Erythrina fusca*	Leaves	Methanol	---	[97]
**74**	Erycristin	*Erythrina fusca*	Leaves	Methanol	---	[97]
**75**	Dolichin A	*Erythrina fusca*	Leaves	Methanol		
		Erythrina subumbrans	Twigs and leaves	Ethanol 95%	---	[100]
**76**	Dolichin B	*Erythrina fusca*	Leaves	Methanol	---	[97]
		Erythrina subumbrans	Twigs and leaves	Ethanol 95%	---	[100]
**77**	Erythbidin D	*Erythrina fusca*	Leaves	Methanol	---	[97]
		Erythrina crista-galli	Leaves and stems	Methanol	---	[105]
		Erythrina stricta	Stem barks	Ethanol 95%	---	[99]
**78**	1-Methoxyerythrabyssin II	*Erythrina fusca*	Leaves	Methanol	---	[97]
		Lespedeza bicolor	Root bark	Methanol	Anti-coronaviral	[78]
**79**	Demethylmedicarpin	*Erythrina fusca*	Leaves	Methanol	---	[97]
**80**	Sandwicarpin	*Erythrina fusca*	Leaves	Methanol	---	[97]
**81**	Erybraedin A	*Erythrina lysistemon*	Root bark	Dichloromethane	---	[96]
**82**	Indigocarpan	*Indigofera aspalathoides*	Stem	Ethyl acetate	Anti-metastatic	[53]
**83**	Apoplanesiacarpan B	*Apoplanesia paniculata*	Whole plant	Ethyl acetate	---	[62]
**84**	Erybreadin B	*Erythrina lysistemon*	Root bark	Dichloromethane	---	[96]
**85**	(6a*R*,11a*R*)-3,8-dihydroxy-9-methoxypterocarpan	*Dalbergia velutina*	Roots	Dichloromethane	---	[106]
**86**	(-)-(6a*R*,11a*R*)-3-hydroxy-9-methoxypterocarpan 8-*O*-α-*D*-ribofuranoside	*Gueldenstaedtia verna*	Whole plant	Methanol:Water	---	[107]
**87**	Erybraedin C	*Bituminaria basaltica*	Aerial parts	Dichloromethane	---	[108]
		Bituminaria bituminosa	Aerial parts	Ethanol:Water	Anti-neuroblastoma	[54]

**88**	Bitucarpin A	*Bituminaria basaltica*	Aerial parts	Chloroform	---	[108]
**89**	Bitucarpan B	*Bituminaria bituminosa*	Aerial parts	Ethanol:Water	__	[54]
**90**	3,8-dimethoxybitucarpin B	*Lonchocarpus eriocalyx*	Stem bark	Dichloromethane: Methanol	---	[104]
**91**	Cabenegrins A-I	*Harpalyce brasiliana*	Roots	Ethanol 95%	---	[48]
**92**	Cabenegrin A-II				Antivenom	[48]
**93**	Harpalycin I-A				---	[48]
**94**	Harpalycin I-C					
**95**	Harpalycin I-D					
**96**	Harpalycin I			Ethanol 95%	---	[48]
**97**	Maackiain	*Millettia brandisiana*	Roots	Dichloromethane	---	[109]
		Harpalyce brasiliana	Roots	Ethanol 95%		[48]
		Ononis angustissima	Roots	Methanol: Water		[103]
		Lonchocarpus bussei	Roots	Dichloromethane: Methanol		[104]
		Millettia pachyloba	Stems	Ethanol: Water		[60]
		Sophora tonkinensis	Roots	Methanol	Anti-inflammatory	[109]
		Gueldenstaedtia verna	Whole plant	Ethanol: Water	---	[107]
**98**	Erysubin E	*Erythrina brucei*	Root bark	Hexane	---	[99]
**99**	Phaseolin	*Erythrina subumbrans*	Twigs and leaves	Ethanol 95%	---	[100]
		Erythrina lysistemon	Root bark	Dichloromethane		[96]
**100**	Vouacapan	*Vatairea macrocarpa*	Root bark	Ethyl acetate	Antifungal	[111]
**101**	Variabilin	*Millettia pachyloba*	Stem	Ethanol: Water	---	[60]
**102**	Pisatin					
**103**	3,9-Dihydroxy-4-prenyl-[6a*R*;11a*R*] pterocarpan	*Bituminaria basaltica*	Aerial parts	Chloroform	---	[108]
**104**	Sandwicensin	*Erythrina fusca*	Leaves	Methanol	---	[97]
**105**	Eryvarin J	*Erythrina brucei*	Root bark	Hexane	---	[98]

**106**	Homopterocarpin	*Pterocarpus erinaceus*	Bark	Ethanol	Antioxidant and Hepatoprotective	[39]
		Erythrina stricta	Stem barks	Ethanol 95%	---	[99]
**107**	4-Hydroxymedicarpin	*Dalbergia velutina*	Roots	Dichloromethane	Cytotoxic	[106]
**108**	Medicarpin 3-*O*-glucoside	*Gueldenstaedtia verna*	Whole plant	Ethanol: Water	---	[107]
**109**	Medicarpin 3-*O*-*β*-*D*-glucopyranoside6'-acetate	*Gueldenstaedtia verna*	Whole plant	Ethanol: Water	---	[107]
**110**	Medicarpin-3-*O*-*β*-*D*-glucoside-6″-*O*-piperidin-2-ylacetat	*Ononis spinosa*	Roots	Methanol: Water	---	[102]
**111a**	(+)-Medicarpin	*Erythrina stricta*	Stem bark	Ethanol 95%	---	[99]
**111 b**	(-)-Medicarpin	*Millettia brandisiana*	Roots	Dichloromethane	---	[109]
		Harpalyce brasiliana	Roots	Ethanol 95%		[48]
		Ononis angustissima	Roots	Methanol: Water		[103]
		Millettia pachyloba	Stems	Ethanol: Water		[60]
		Dalbergia velutina	Roots	Dichloromethane		[106]
		Gueldenstaedtia verna	Whole plant	Ethanol: Water		[107]
		Erythrina crista-galli	Leaves and stems	Methanol		[105]
**112**	Sophoratonkin	*Sophora tonkinensis*	Roots	Methanol	Anti-inflammatory	[109]
**113**	6a-Hydroxymedicarpin	*Sophora flavescens*	Roots	Ethanol		[112]
**114**	Eryvarin K	*Erythrina lysistemon*	Root bark	Dichloromethane		[96]
**115**	Apoplanesiacarpan A	*Apoplanesia paniculata*	Whole plant	Ethanol	---	[62]
**116**	Melilotocarpan C	*Apoplanesia paniculata*	Whole plant	Ethanol	Antiplasmodial	[62]
**117**	Hydroxycristacarpone	*Erythrina fusca*	Leaves	Methanol	---	[97]

**118**	3H-Benzofuro[3,2-c][1]benzopyran-3-one, 6,6a,11a,11b-tetrahydro-6a,11b-dihydroxy-9-methoxy-, (6aS,11aR,11bS)- (ZCI)	*Sophora flavescens*	Roots	Ethanol	---	[112]
**119**	Velucarpin A	*Dalbergia velutina*	Roots	Dichloromethane	Cytotoxic	[106]
**120**	Velucarpin B					
**121**	Velucarpin C					
**122**	Erybraedin D	*Bituminaria bituminosa*	Aerial parts	Ethanol:Water	---	[54]
**123**	Lespebuergine G4	*Lespedeza bicolor*	Root bark	Methanol	Anti-coronaviral	[63]
**124**	(-)-Maackiain-3-*O*-glucoside (Trifolirhizin)	*Ononis angustissima*	Aerial parts	Methanol:Water	---	[113]
			Roots			[103]
		Gueldenstaedtia verna	Whole plant	Methanol:Water		[107]
**125**	Bicolosin A	*Lespedeza bicolor*	Root bark	Methanol	Anti-coronaviral	[63]
**126**	Bicolosin B					
**127**	Bicolosin C				---	
**128**	1-Methoxyphaseollidin	*Erythrina stricta*	Stem bark	Ethanol 95%	---	[99]
**129**	Erystrictins A					
**130**	Erystrictins B					
**131**	Erystrictins C					
**132**	Erystrictins D	*Erythrina subumbrans*	Twigs and leaves	Ethanol 95%	---	[120]
		Erythrina stricta	Stem bark			[99]
**133**	Fuscacarpan B	*Erythrina stricta*	Stem bark	Ethanol 95%	---	[121]
**134**	Fuscacarpan C					
**135**	Bituminarin A	*Bituminaria bituminosa*	Aerial parts	Ethanol:Water	---	[121]
**136**	Bituminarin B					
**137**	Bituminarin C					
**138**	Eryvarin E	*Erythrina variegata*	Root bark	Acetone	---	[95]

Table S7. Rotenoids

N°	Compound	Species	Plant part	Extract solvent	Activity	Reference
**139**	(±)-Villosinol	*Millettia brandisiana*	Roots	Dichloromethane	Cytotoxic	[109]
**140**	(-)-cis-12a-Hydroxyrotenone					
**141**	(-)-Tephrosin	*Millettia brandisiana*	Roots	Dichloromethane	Cytotoxic	[109]
		Millettia oblata ssp. teitensis	Leaves	Methanol: Dichloromethane	Antiviral	[114]
**142**	α-Toxicarol	*Millettia brandisiana*	Leaves	Ethyl acetate	Cytotoxic	[115]
**143**	12a-hydroxy-α-toxicarol	*Millettia brandisiana*	Leaves	Ethyl acetate	Cytotoxic	[115]
**144**	Dalbinol	*Millettia pachyloba*	Stems	Ethanol	Cytotoxic	[80]
**145**	Pongarotene	*Millettia pinnata*	Seeds	Methanol	Larvicidal	[116]
**146**	Stemonone	*Clitoria fairchildiana*	Roots	Methanol	---	[9]
**147**	Stuhlmarotenoid A	*Xeroderris stuhlmannii*	Leaves	Ethanol	Antibacterial	[117]
**148**	Stuhlmarotenoid B					
**149**	Stuhlmarotenoid C					
**150**	6a*S*, 12a*S*, 12*S*-elliptinol	*Millettia pyrrhocarpa*	Leaves and twigs	Hexane	Antimicrobial and Cytotoxic	[118]
**151**	6a*S*, 12a*S*, 12*S*-munduserol					
**152**	Dehydromunduserone					
**153**	(–)-(6a*R*,12a*R*)-Millettiabrandisin A	*Millettia brandisiana*	Leaves	Ethyl acetate	Cytotoxic	[115]
**154**	(–)-(6a*R*,12a*R*)-Millettiabrandisin B					
**155**	(–)-(6a*R*,12a*R*)-Millettiabrandisin C					
**156**	(–)-(6a*S*,12a*S*)-6-deoxyclitoriacetal					
**157**	Sermundone					
**158**	6a,12a-dehydrosurmundone					

**159**	6a,12a-dehydro-α-toxicarol					
**160**	Oblarotenoid E	*Millettia oblata ssp. teitensis*	Leaves	Methanol: Dichloromethane	Antiviral	[114]
**161**	Oblarotenoid F					
**162**	Oblarotenoid G					
**163**	Oblarotenoid C					
**164**	Oblarotenoid A					
**165**	Oblarotenoid D					
**166**	12a-hydroxymunduseron	*Millettia caerulea*	Fruits	Methanol	---	[119]
**167**	Deguelin					
**168**	(−)-Caeruleanone D					
**169**	(−)-3-Deoxycaeruleanone D					
**170**	(−)-3-Hydroxycaeruleanone A					
